# Changes in motor behavior and lumbar motoneuron morphology following repeated chlorpyrifos exposure in rats

**DOI:** 10.1371/journal.pone.0305173

**Published:** 2024-06-14

**Authors:** Shannon H. Romer, Kaitlyn M. Miller, Martha J. Sonner, Victoria T. Ethridge, Nathan M. Gargas, Joyce G. Rohan

**Affiliations:** 1 Environmental Health Effects Laboratory, Naval Medical Research Unit Dayton, Wright-Patterson AFB, Dayton, OH, United States of America; 2 Leidos, Reston, VA, United States of America; 3 Oak Ridge Institute for Science and Education, Oak Ridge, TN, United States of America; 4 Department of Neuroscience, Cell Biology and Physiology, Wright State University, Dayton, OH, United States of America; Weizmann Institute of Science, ISRAEL

## Abstract

Chlorpyrifos is an organophosphate pesticide associated with numerous health effects including motor performance decrements. While many studies have focused on the health effects following acute chlorpyrifos poisonings, almost no studies have examined the effects on motoneurons following occupational-like exposures. The main objective of this study was to examine the broad effects of repeated occupational-like chlorpyrifos exposures on spinal motoneuron soma size relative to motor activity. To execute our objective, adult rats were exposed to chlorpyrifos via oral gavage once a day, five days a week for two weeks. Chlorpyrifos exposure effects were assessed either three days or two months following the last exposure. Three days following the last repeated chlorpyrifos exposure, there were transient effects in open-field motor activity and plasma cholinesterase activity levels. Two months following the chlorpyrifos exposures, there were delayed effects in sensorimotor gating, pro-inflammatory cytokines and spinal lumbar motoneuron soma morphology. Overall, these results offer support that subacute repeated occupational-like chlorpyrifos exposures have both short-term and longer-term effects in motor activity, inflammation, and central nervous system mechanisms.

## Background

Chlorpyrifos (CPF) is an organophosphate (OP) pesticide that was a widely used on crops in the USA [1]. Despite successful efforts to reduce the use of CPF in the USA over the last decade, a ruling by the U.S. Court of Appeals for the Eight Circuit in November of 2023 officially permits the use of CPF on crops, reversing the U.S. Environmental Protection Agency’s 2021 ban [2,3]. CPF is considered hazardous to humans and acute exposures have been associated with neurological effects, autoimmune diseases, developmental disorders, and even death. High levels of OP exposure, or poisonings, inhibit acetylcholinesterase (AChE) enzyme activity and, in these situations, can lead to cholinergic crisis causing paralysis and respiratory failure [4]. Additionally, OP poisoning is associated with delayed-onset health effects in humans such as intermediate syndrome, OP-induced delayed polyneuropathy, and chronic OP-induced neuropsychiatric disorder [5–8].

While higher concentration exposures have been heavily studied, there is compelling evidence that repeated lower concentration OP exposures, such as those that may occur occupationally or in environments where OP pesticides are repeatedly or regularly applied, may not severely impact AChE activity but may still be linked to adverse neurological effects in humans and animals [9–14]. Neurological impairments include deficits in attention span, information processing speeds, motor control and motor coordination [14,15]. Farmworkers, in particular, have been shown to have neurological effects associated with the presence of systemic OP metabolites [16–21]. Furthermore, multiple neurodegenerative diseases appear to be associated with repeated exposure to OPs [22,23]. Thus, there is a need to better understand the impacts of occupational-like or repeated low-level OP exposure that can lead to health effects or neurological deficits.

High concentration OP pesticide poisonings appear to be associated with motoneuron (MN) decrements and decreases in motor activity [23]. To our knowledge, no studies have examined the effects of occupational-like (i.e. repeated) exposures to CPF on MN structure and function. The goal of this study was to examine the broad effects of repeated CPF exposures on MN structure relative to overall motor activity. We exposed Sprague Dawley rats to once daily CPF via gavage five times a week for two weeks (10 days total) and examined effects at three days and two months following the last exposure. Our results revealed that repeated occupational-like exposure to CPF had both immediate and delayed effects. Overall, we did not find evidence of neurodegeneration at either time point as indicated by either a loss of MNs or MN atrophy. We did, however, find that repeated CPF exposure resulted in transient effects in open-field motor activity. We also demonstrate delayed effects in sensorimotor integration, increased expression of pro-inflammatory cytokines, and enlargement of spinal lumbar MNs.

## Materials and methods

### Animals and exposures

The animals involved in this study were procured, maintained, and used according to an Institutional Animal Care and Use Committee (IACUC)-approved Animal Use Protocol (FWA-2018-0174A) and established animal welfare standards, compliant with: DoD Instruction 3216.01 (DoD, 2019); U.S. Department of Agriculture Animal Welfare Act and Regulations (USDA-APHIS *Blue Book*, 2023); Defense Health Agency Multi-Service Regulation (DHA-MSR) 6025.02, and *The Guide for the Care and Use of Laboratory Animals*, 8th Edition, National Research Council (2011). Wright-Patterson Air Force Base (WPAFB) Wright-Patterson, Ohio has been accredited by AAALAC International since 1966. A total of 108 adult male Sprague Dawley rats from Charles River Laboratories (Wilmington, MA, USA) were used in this study. CPF exposure impacts rodent behavior in a sex dependent manner. Male rats were chosen to reduce sex-dependent variability because they appear to have the more dramatic reduction of cholinesterase activity following CPF exposure [24]. Future studies are required to determine sex-dependent differences in the effects reported herein. Rats were maintained with a 12 hour light/dark cycle and provided dry chow and water *ad libitum* in a temperature (20 to 26°C) and humidity (30 to 70%) controlled vivarium. For rat exposures, CPF (Cat # N-11459, Chem Service Inc., West Chester, PA, USA) was diluted in corn oil and administered via gavage at 1.0 mL/kg body weight (bw). CPF exposures occurred once a day, five days per week, for two weeks for a total of 10 exposure days to simulate occupational exposures. There were three different exposure groups: 0.0 mg CPF/kg*bw (corn oil only), 5.0 mg CPF/kg *bw, and 10.0 mg CPF/kg*bw. CPF exposure concentrations were selected based on previous studies demonstrating neurological effects from repeated oral exposures [25–27]. There were two time points for endpoint analyses. The immediate time point was analyzed 3 days following the last exposure. The delayed time point was analyzed 2 months following the last exposure. Rats were randomly assigned to one of the six experimental groups and each experimental group contained eighteen rats. Twelve rats per group were used both for behavior tests and pro-inflammatory cytokine assessments. Six rats per group were used for immunohistochemistry and microscopy. Blood collected from all eighteen rats per experimental group was used for the acetylcholinesterase activity assay. Rats were euthanized via exsanguination during transcardial perfusions (see immunohistochemistry methods) under deep anesthesia (100–150 mg/kg*bw of sodium pentobarbital).

### Cholinesterase activity assay

Cholinesterase (ChE) activity levels were analyzed at both the immediate and delayed time points following the repeated exposures to CPF. Blood was collected during terminal procedures and processed to collect plasma and plasma was stored in -80°C freezer. A commercially available AChE enzyme-linked immunosorbent assay (ELISA) Kit (LifeSpan Biosciences, Seattle, WA, USA) was then used to quantify plasma activity levels.

### Neurobehavior

Motor Activity: Motor activity was analyzed using clear 16” x 16” polycarbonate open fields with an automated Photobeam Activity System (PAS, San Diego Instruments, San Diego, CA, USA). Two sets of stacked photobeams were used to track both horizontal and vertical movements. The proprietary software (PAS, San Diego Instruments, San Diego, CA, USA) tracked and quantified both fine movements (one photobeam break) and locomotor movements (two photobeams broken in sequence). Recordings occurred for 30 minutes under controlled conditions including noise, temperature, humidity, lighting, odors, and environmental distractions. Quantified endpoints included distance traveled, active time, total rears, fine movements, percentage of time in the center of the arena, and locomotor speed.

Prepulse Inhibition (PPI) of Acoustic Startle Response: The acoustic startle reflex test was performed to assess sensorimotor gating in a SR-Lab system (San Diego Instruments, San Diego, CA, USA). Prior to testing, the chambers were calibrated for noise levels in decibels (dB) of sound pressure level (SPL) via a sound meter as well as startle sensor sensitivity. For testing, rats were placed in an acrylic cylinder located inside a sound-attenuated test chamber. Following a 5-minute acclimation, the rats were then tested using a 4 x 4 Latin square sequence of trials including 1) no stimulus (65 dB SPL background white noise), 2) a 120 dB SPL startle stimulus with no prepulse, 3) a 75 dB SPL prepulse with 120 dB SPL startle, or 4) an 85 dB SPL prepulse with 120 dB SPL startle. The sequence of trials was repeated 6 times for a total of 106 trials lasting approximately 25 minutes. Trials of the same type are averaged together. The intertrial interval is randomized in the range of 5–15 sec. Prepulse stimulus duration was 20 ms and the startle signal was 40 ms. The interval between prepulse and startle signals was 70 ms. Startle amplitude was measured by a piezoelectric accelerometer located below the acrylic cylinder and calculated as integrated response of the whole-body startle reaction over 100 ms. Percent PPI was calculated as a percent reduction of the startle amplitude (A):

$$\%PPI=100-\frac{A_{Startle\;}-A_{\mathrm{Pr}epulse\;+\;Startle\;}}{A_{Startle\;}}\times 100$$

### Pro-inflammatory cytokines

Cytokines were measured in blood plasma collected during terminal procedures at the immediate and delayed time points. Multiple inflammatory cytokines (IFN-γ, IL-1β, IL-4, IL-5, IL-6, IL-10, IL-13, KC/GRO, and TNF-α) were measured using a multi-spot electrochemiluminescent array (Meso Scale Discovery, Rockville, MD, USA). Samples were prepared and analyzed per manufacturer instructions. All cytokine measurements were performed in duplicate and data with a coefficient of variation greater than 30 were omitted.

### Immunohistochemistry and microscopy

**Fig 1:**
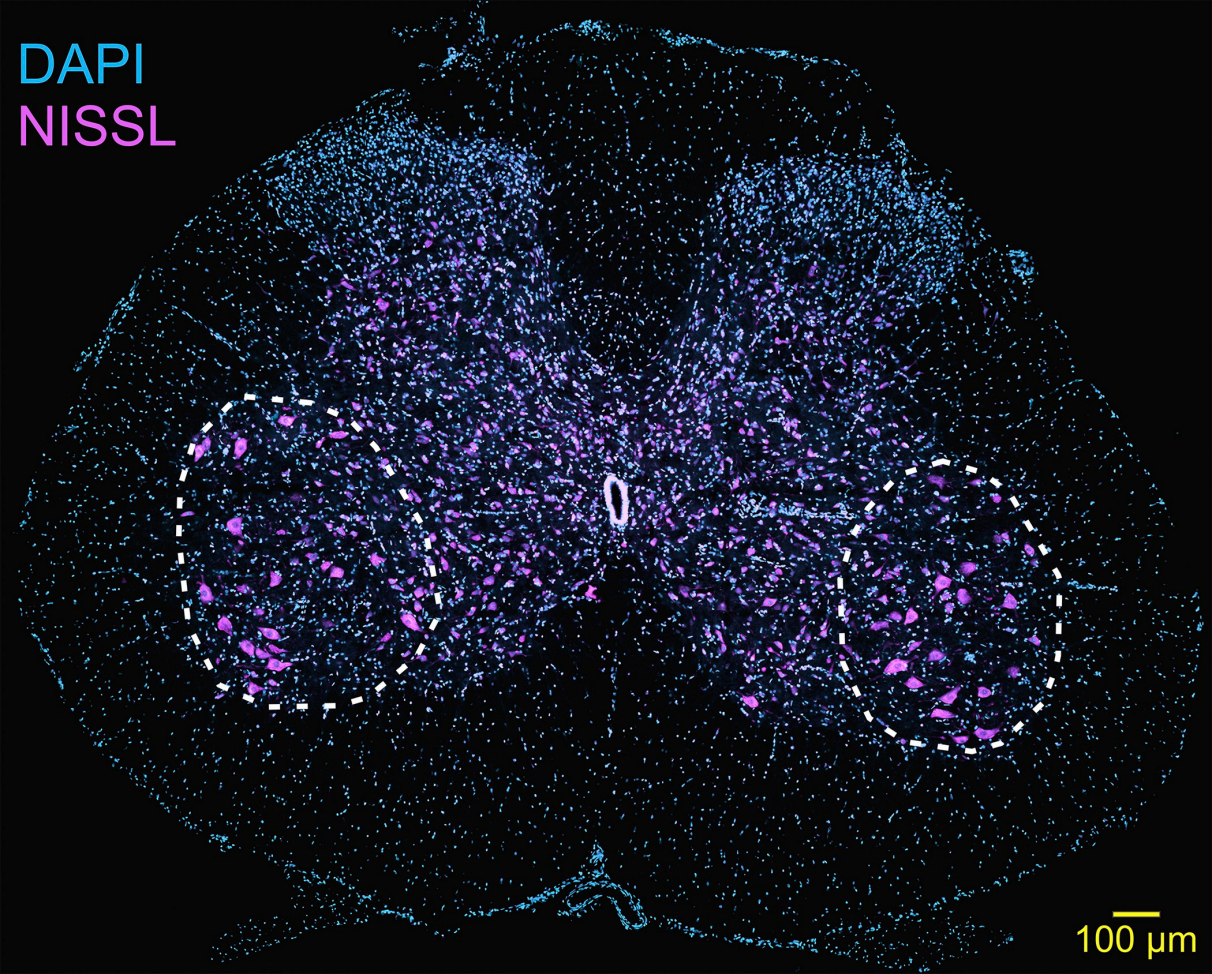
Rat lumbar spinal motoneurons. Confocal micrograph of transverse L5 spinal cord slice with DAPI (blue) staining to reveal nuclei and Nissl (purple) staining to reveal neurons. MNs selected for analysis were localized in lateral lamina IX of the ventral horn shown in the white encircled regions. Scale Bar = 100 μm.

To prepare tissue for immunohistochemistry and microscopy analysis, rats were transcardially perfused with 4% paraformaldehyde in 0.1M phosphate buffer. The spinal cords were then removed and placed into 4% paraformaldehyde for two hours then cryoprotected overnight in 15% sucrose solution. The lumbar spinal cords were sectioned into 70 micrometer transverse slices on a cryostat (Leica Biosystems, Wetzler, Germany). Spinal cord slices from lumbar level 5 were stained floating. Neurons were labeled using green fluorescent Nissl staining (Invitrogen/Thermo Fisher, Carlsbad, CA, USA, Catalogue #N21480) diluted 1:500 in a PBS buffer with 0.1% Triton X-100. The tissue was then mounted on microslides in VECTASHIELD media (Vector Laboratories, Newark, CA, USA). Using the Leica SP8 confocal microscope (Leica Microsystems, Deerfield, IL, USA), the tissue was imaged with a 20x objective lens at 10 x 1.0 μm z-step with solid state lasers. Multiple images were acquired to cover the region of Lamina IX in the ventral horn of the spinal cord where MNs are localized. Consistent with other studies, MNs selected for quantification were differentiated from other ventral horn neurons by morphology and laminar location [28–31]. Specifically, sampled neurons were localized in lateral lamina IX of the spinal cord, with larger diameters, and grouped into motor pools with similar soma shapes compared to other neurons in nearby laminae. The individual images were stitched together and MNs were quantified. The MNs in lamina IX were quantified by counting the nuclei and measuring the soma mean diameters at the level of the nucleolus in LasX software (Leica Microsystems, Deerfield, IL, USA). A total of six spinal cord sections (three right and three left) were selected per rat for MN quantification (Fig 1). Due to poor preservation quality, two spinal cords were eliminated from microscopy.

### Statistics

All procedures and analyses were performed by individuals blinded to the exposure groups. All statistical tests were performed in SigmaPlot software (Systat, Santa Clara, CA, USA). Comparisons for AChE activity, cytokine assays, and MN measurements was made using one-way analysis of variance (ANOVA) after data passed tests for normality and equal variance. Post-hoc pairwise comparison procedures (Holm-Sidak method) were used to determine significance in individual treatment groups compared to controls. To determine statistical differences in open field motor activity, two-way ANOVA was used followed by post-hoc pairwise comparison procedures versus control (Dunnett’s method). Normality tests failed for acoustic startle studies and some of the cytokine assays, therefore ANOVA on ranks was used with Tukey’s test for pairwise comparisons with 0 mg CPF/kg exposure controls. Statistical significance was accepted at p< 0.05. Values are reported as mean ± SEM in graphs and mean ±SD in text and tables.

## Results

### Cholinesterase activity is reduced following repeated chlorpyrifos exposures

**Fig 2:**
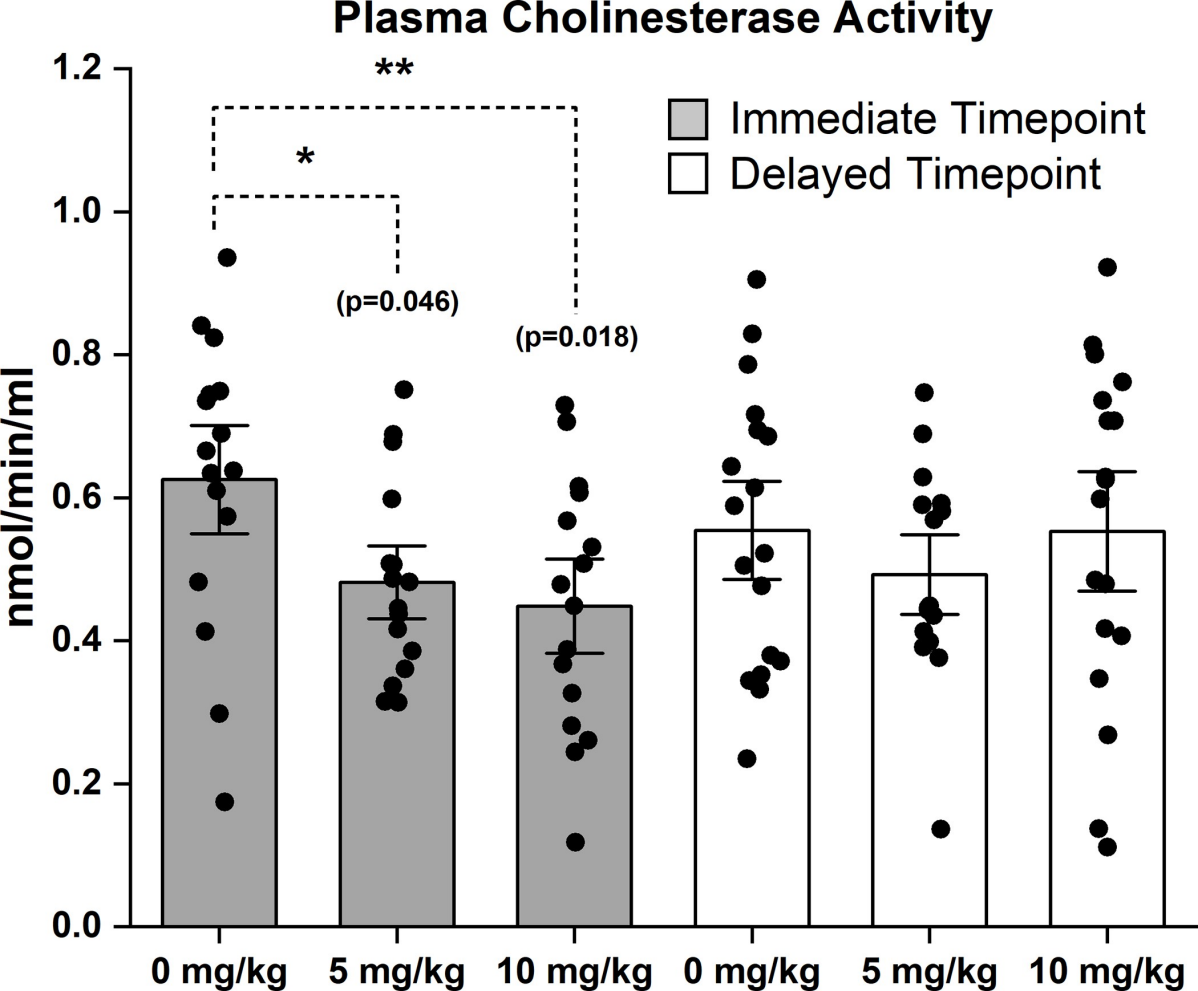
Transient reduction in plasma cholinesterase (ChE) activity following repeated exposure to 5 mg CPF/kg and 10 mg CPF/kg. Plasma ChE activity levels were quantified using ELISA on plasma collected either 3 days or 8 weeks following the last exposure for the immediate and the delayed groups, respectively. One-way ANOVA revealed significant differences at the immediate time point (p = 0.014) and no significant differences at the delayed time point. For the immediate time point, n = 16 for each exposure group. For the delayed time point, n = 18 for the 0 mg/kg and 10 mg CPF/kg exposure groups and n = 16 for the 5 mg CPF/kg exposure group. Error bars = ±SEM.

Blood plasma was collected following exposures to CPF at either 3 days (the immediate group) or 8 weeks (the delayed group) after the final exposure. A commercially available kit was used to monitor AChE activity (S1 Table), however, the kits may also be detecting activity from other plasma cholinesterases, such as butyrylcholinesterase. In the immediate group, there was a significant 23% reduction in ChE activity in the 5 mg CPF/kg exposure group compared to controls (0 mg CPF/kg: 0.625 mmol/min/mL ± 0.2 SD vs. 5 mg CPF/kg: 0.482 mmol/min/ml ± 0.1 SD, p = 0.046, ANOVA, Holm-Sidak method) (Fig 2 and S1 Table). Also in the immediate group, there was a significant 28% reduction in ChE activity in the 10 mg CPF/kg exposure group compared to controls (10 mg CPF/kg: 0.449 mmol/min/mL ± 0.2 SD, p = 0.018 ANOVA, Holm-Sidak method).While reductions were evident 3 days following the last exposure, there were no significant differences between the different exposure groups approximately two months later (0 mg CPF/kg: 0.554 mmol/min/mL ± 0.2 SD vs. 5 mg CPF/kg: 0.493 mmol/min/mL ± 0.1 SD vs. 10 mg CPF/kg: 0.553 mmol/min/mL ± 0.2 SD, p = 0.594 ANOVA) (Fig 2 and S1 Table). Overall, the effect of CPF exposure on ChE activity was dose-dependent and transient.

### Transient effects in open field motor activity following repeated chlorpyrifos exposures

Open field motor activity was used to assess general locomotor and behavior activity levels in the exposed rats. Three days following the last CPF exposure, there were significance decreases in total locomotion as measured by the number of photobeam breaks that occurred during open field exploration at both exposure levels (Table 1). There were also significant decreases in the total active time and the total distance traveled in CPF exposed rats (Table 1). Vertical rearing behavior did not change following CPF exposure neither did the amount of time the rats spent in the center of the field. Raw motor activity data is in S2 Table. These behaviors suggest that there were no anxiety enhancing effects. With regard to the delayed group (8 weeks following the last CPF exposure), there were no significant effects on open field motor activity (Table 1).

**Table 1:** Open field motor activity effects following repeated chlorpyrifos exposure.

Motor Activity Parameters	Daily Chlorpyrifos Exposure Doses			ANOVA p-Value
	0 mg/kg	5 mg/kg	10 mg/kg	
Three Days Post Last Exposure				
Locomotion (# Beam Breaks)	5,456 ± 1,117	4,150 ± 931* (p = 0.009)	4,240 ± 1,104* (p = 0.015)	0.007
Fine Movements (# Stereotypical Beam Breaks)	236.7 ± 17.4	233 ± 34.6	232 ± 24.1	0.90
% Time in Center	41.6 ± 4.7	42.0 ± 5.5	43.2 ± 5.3	0.75
Total Rears	164.4 ± 77.6	145.0 ± 96.0	138.2 ± 71.1	0.72
Speed (cm/sec)	36.4 ± 2.4	34.7 ± 2.4	34.7 ± 2.4	0.15
Active Time (sec)	1,354 ± 156	1,124 ± 185* (p = 0.004)	1,226 ± 156	0.007
Total Distance (cm)	31,384 ± 6,606	24,274 ± 5,677* (p = 0.01)	26,378 ± 5,388 (p = 0.08)	0.02
Eight Weeks Post Last Exposure				
Locomotion (# Beam Breaks)	4,808 ± 1,341	5,133 ± 1,474	4,624 ± 1,444	0.68
Fine Movements (# Stereotypical Beam Breaks)	201.3 ± 20.1	220.4 ± 26.2	209.0 ± 21.1	0.13
% Time in Center	50.7 ± 4.5	50.2 ± 6.1	49.2 ± 5.8	0.79
Total Rears	112.2 ± 39.8	126.9 ± 45.0	121.9 ± 36.6	0.67
Speed (cm/sec)	40.3 ± 3.2	41.0 ± 3.7	40.0 ± 4.8	0.81
Active Time (sec)	1,186 ± 195	1,245 ± 221	1,211 ± 162	0.75
Total Distance (cm)	30,870 ± 7,958	33,620 ± 9,336	30,720 ± 8,560	0.66

*Indicates statistical significant difference from control (0 mg/kg) with p-value calculated with post-hoc comparisons (Dunnett’s method). N = 12 per group, mean ± SD.

### Delayed sensorimotor gating effects following repeated exposure to chlorpyrifos

The acoustic startle response is an exaggerated jumping response caused by involuntary whole-body muscle contraction that occurs following an unexpected loud auditory stimulus. This startle response can be attenuated when it is preceded by a weaker prepulse auditory stimulus [32,33]. Impairment in prepulse inhibition (PPI) of acoustic startle responses is linked to dysfunction in sensorimotor gating function in humans and rodents [34–40]. PPI of acoustic startle was used in this study as an operational measure of sensorimotor gating. Detailed acoustic startle data is listed in S3 Table.

**Fig 3:**
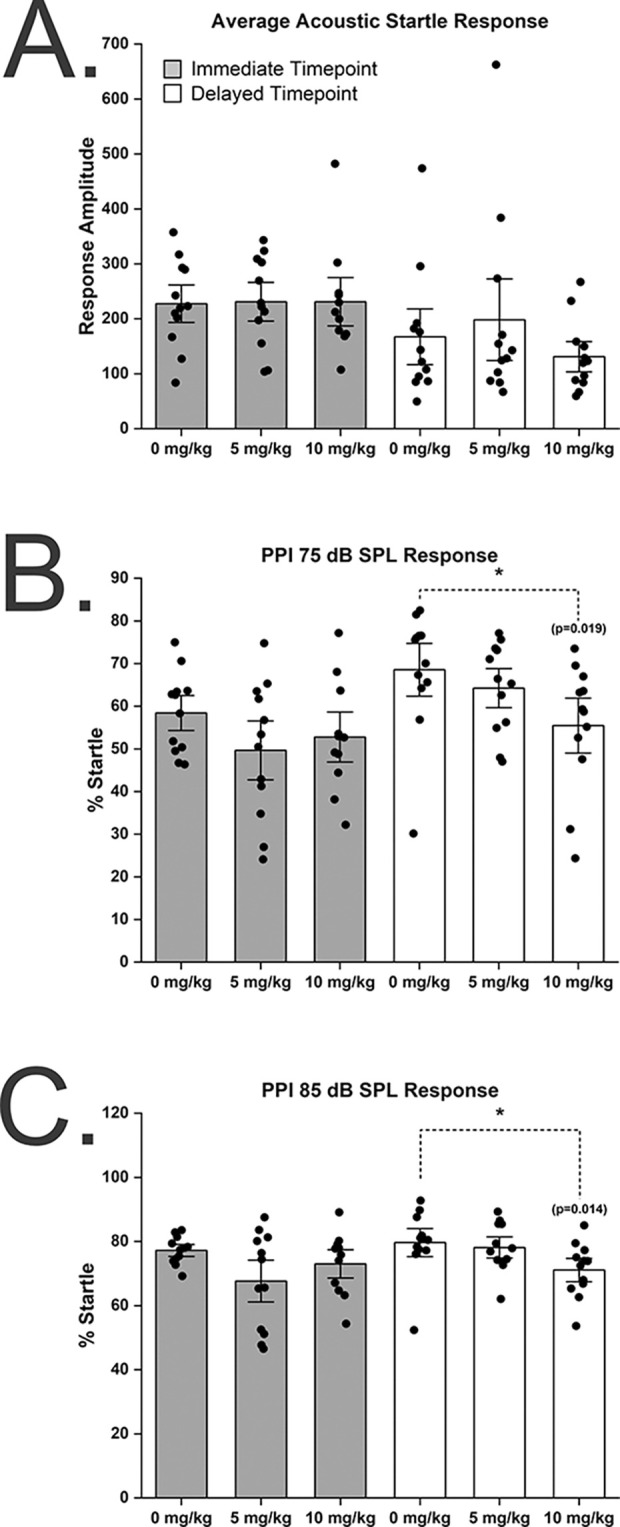
Prepulse Inhibition (PPI) of acoustic startle response decreased two months after repeated chlorpyrifos daily doses. 65 dB SPL background white noise was used in all rats tested. The amplitude of the startle response was measured with a piezoelectric accelerometer in millivolts. All tests were performed either 3 days following the last CPF exposure for the immediate group and 8 weeks following the last CPF exposure for the delayed group. Repeated CPF concentrations shown in figure apply to all graphs. **A**. Acoustic startle response with no prepulse was measured following a 120 dB SPL acoustic stimulus. Significant differences were not found at the immediate or delayed time points. **B.** Acoustic startle responses recorded after a 75 dB SPL prepulse followed by a 120 dB SPL startle stimulus. Significant differences were only detected at the delayed time point. **C.** Acoustic startle responses recorded after an 85 dB SPL prepulse followed by a 120 dB SPL startle stimulus. Significant differences were only detected at the delayed time point. For the immediate time point, n = 12 for the 0 mg/kg and 5 mg/kg groups and n = 11 for the 10 mg/kg group. For the delayed time point, n = 12 for all groups. Asterisks denote significant differences compared to 0 mg/kg controls as determined with ANOVA post-hoc Tukey’s test. Error bars = ± SEM.

Three days after the last exposure to CPF, there were no significant differences in the average acoustic startle amplitude (0 mg CPF/kg: 227.5 mv ± 79.0 SD vs. 5 mg/kg: 231.1 mv ± 81.5 mv SD vs. 10 mg/kg: 231.0 ± 97.6 SD, p = 0.99 ANOVA) (Fig 3A) and in the PPI at both the 75 dB prepulse (0 mg CPF/kg: 58.4 ± 9.5 SD vs. 5 mg/kg: 49.6 ± 15.9 SD vs. 10 mg/kg: 52.8 ± 13.0 SD, p = 0.26 ANOVA) and 85 dB prepulse (0 mg CPF/kg: 77.2 ± 4.3 SD vs. 5 mg/kg: 67.7 ± 15.0 SD vs. 10 mg/kg: 73.0 ± 9.8 SD, p = 0.10 ANOVA). Two months after the last CPF exposure there continued to be no significant changes in the average acoustic startle amplitude (0 mg CPF/kg: 167.3 ± 116.8 SD vs. 5 mg/kg: 198.3 ± 49.5 SD vs. 10 mg/kg: 131.1 ± 63.8 SD, p = 0.43 ANOVA) (Fig 3B and 3C). There were, however, significant differences at the delayed time point in the PPI at both the 75 dB prepulse (0 mg CPF/kg: 68.5 ± 14.3 SD vs. 5 mg/kg: 64.2 ± 10.6 SD vs. 10 mg/kg: 55.5 ± 14.9 SD, p = 0.03 ANOVA) (Fig 3B) and 85 dB prepulse (0 mg CPF/kg: 79.6 ± 10.0 SD vs. 5 mg/kg: 78.1 ± 7.6 SD vs. 10 mg/kg: 71.1 ± 8.4 SD, p = 0.05 ANOVA) (Fig 3C). The significant differences at the delayed time point were only between the highest dose and vehicle control groups.

### Systemic pro-inflammatory cytokines are elevated two months following repeated chlorpyrifos exposure

**Fig 4:**
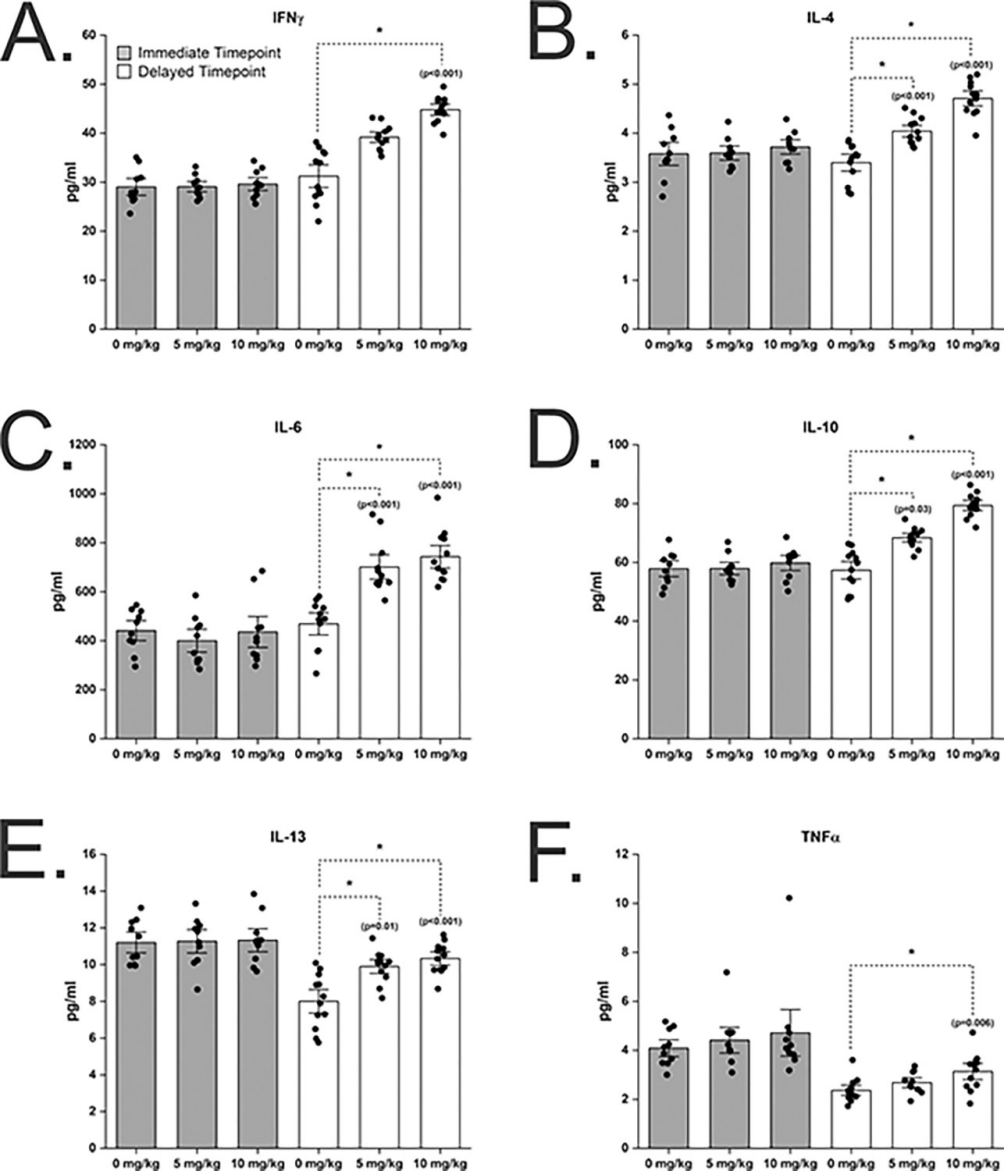
Pro-inflammatory cytokines are increased in plasma 2 months following repeated chlorpyrifos exposure. Repeated CPF concentrations shown in figure apply to all graphs. No significant differences were identified at the immediate time point (3 days following the last exposure). Significant differences were identified at the delayed time point (2 months following the last exposure). Significant differences compared to controls (0 mg CPF/kg) shown were calculated using the post-hoc pairwise comparison tests Holm-Sidak method for parametric data sets and the Tukey’s method for nonparametric data sets. All significant p-values are shown in the figure. N = 10 per group for immediate time point and n = 12 per group for delayed time point with the exception includes n = 10 for TNFα 5 mg CPF/kg exposure group. Specific statistical comparison tests used are A) Tukey B) Holm-Sidak C) Holm-Sidak D) Tukey’s E) Tukey’s F) Holm-Sidak. Error bars = ± SEM.

To evaluate the effect of repeated exposure to CPF on systemic inflammation, a nine-multiplex cytokine array was performed on plasma collected either three days (immediate) or 2 months (delayed) following the last CPF exposure (Table 2, Fig 4). Raw data used to generate means and figures are listed in S4 Table. For IL-1β and IL-5 cytokines, signal was not detected in the samples. No significant differences were found in plasma pro-inflammatory cytokine levels three days following the last exposure for IFNγ, IL-4, IL-6, IL-10, IL-13, and TNFα (Table 2, Fig 4). At the delayed time point, significant differences were detected in IFNγ, IL-4, IL-6, IL-10, IL-13, and TNFα in a dose-dependent manner (Table 2 and Fig 4). No significant changes were found in KC-GRO cytokine levels at the delayed time point (Fig 4).

**Table 2:** Plasma pro-inflammatory cytokine concentrations following repeated chlorpyrifos exposure.

Cytokine (pg/mL)	Three Days Following Last CPF Exposure (Immediate)				Two Months Following Last CPF Exposure (Delayed)			
	0.0 mg/kg	5.0 mg/kg	10.0 mg/kg	ANOVA p-Values	0.0 mg/kg	5.0 mg/kg	10.0 mg/kg	ANOVA p-Values
IFNγ	29.0 ± 3.6	29.1 ± 2.2	29.6 ± 2.8	0.89^†^	31.2 ± 5.3	39.2 ± 2.5	44.8 ± 2.7	<0.001‡*
IL-4	3.58 ± 0.50	3.60 ± 0.30	3.72 ± 0.31	0.67^†^	3.40 ± 0.40	4.04 ± 0.28	4.71 ± 0.36	<0.001^†^*
IL-6	441.5 ± 86.3	400.4 ± 97.7	435.2 ± 99.8	0.66^†^	469.2 ± 99.8	701.2 ± 110.2	742.8 ± 105.3	<0.001^†^*
IL-10	57.8 ± 5.7	57.9 ± 4.5	59.8 ± 5.4	0.64^†^	57.3 ± 6.9	68.4 ± 3.4	79.3 ± 4.0	<0.001‡*
IL-13	11.21 ± 1.19	11.27 ± 1.34	11.33 ± 1.33	0.98^†^	8.00 ± 1.47	9.90 ± 0.86	10.34 ± 0.84	<0.001‡*
TNFα	4.09 ± 0.73	4.42 ± 1.11	4.72 ± 2.00	0.87^†^	2.36 ± 0.49	2.68 ± 0.44	3.14 ± 0.76	0.01^†^*
KC-GRO	208.7 ± 111.8	182.9 ± 76.0	194.0 ± 81.3	0.94^†^	147.1 ± 42.4	136.7 ± 54.0	168.0 ± 75.2	0.43^†^

Cytokine plasma concentrations displayed in table are pg/mL.

†Indicates statistical tests on parametric data sets determined with ANOVA

‡Indicates statistical differences identified on nonparametric data sets determined with Kruskal-Wallis ANOVA on Ranks.

* Indicates statistical differences. Overall n = 10 per group for immediate time point and n = 12 per group for delayed time point with the exception of n = 10 for TNFα 5 mg CPF/kg exposure group at the delayed time point. ±SD.

### Lumbar motoneuron soma sizes are altered following repeated exposure to chlorpyrifos

**Fig 5:**
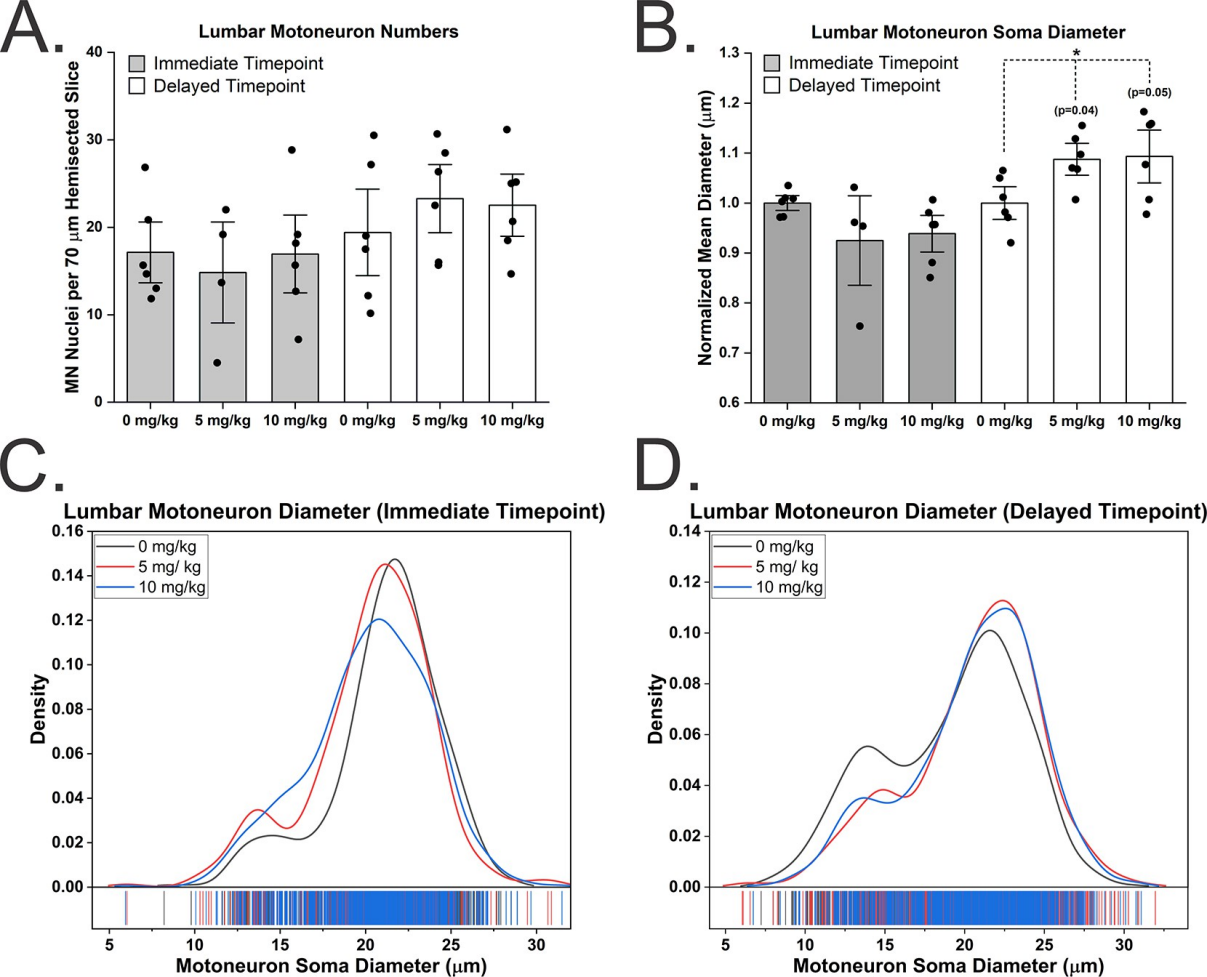
Spinal lumbar motoneuron soma diameters are larger two months following repeated exposure to chlorpyrifos. MNs were quantified in confocal micrographs with z = 1.0 μm steps. Repeated CPF concentrations shown in figure apply to graphs A and B. A) No significant differences were found in the number of MNs at spinal level 5 in unilateral lateral lamina IX. B) MN soma diameters were significantly increased 2 months following the last CPF exposures. C) Distribution of MN soma sizes showed some dedifferentiation of the bimodal distribution in the 10 mg CPF/kg exposure group. D) Distribution of MN soma sizes revealed no shifts in the first mode representing smaller MNs, but a shift in the second mode representing the larger MNs. Error bars = ± SEM.

To determine if spinal MNs are impacted by repeated exposure to CPF, neuron numbers (S5 Table) and soma sizes (S6 and S7 Tables) were measured unilaterally in confocal images collected at 1.0 μm z-steps from 70 μm thick transverse spinal cord slices. An average of 107 MN nuclei (range 95–128) were counted at spinal lumbar level 5 per rat for a total of 3,651 MNs (Fig 5). No significant differences (ANOVA p = 0.86) in the average number of lumbar MNs per unilateral tissue slice were identified 3 days following the last CPF exposure (0 mg CPF/kg: 17.1 ± 5.7 SD vs. 5 mg CPF/kg: 14.8 ± 3.9 SD vs. 10 mg CPF/kg: 16.9 ± 3.0 SD), (Fig 5A). Likewise, no significant differences (ANOVA p = 0.59) were identified 2 months after the last CPF exposure (0 mg CPF/kg: 19.4 ± 8.1 SD vs. 5 mg CPF/kg: 23.3 ± 2.6 SD vs. 10 mg CPF/kg: 22.5 ± 2.4 SD), (Fig 5A).

To determine if repeated exposure to CPF affects the soma size of MNs, the mean diameter was measured in single optical confocal sections through the nucleolus of each MN. An average of 115 MN somas were measured per rat. MN diameters were normalized to control diameters to determine relative change in diameter. No significant differences (ANOVA p = 0.21) in the mean diameter of MN somas were identified 3 days following the last CPF exposure (0 mg CPF/kg: 1.00 ± 0.02 SD vs. 5 mg CPF/kg: 0.93 ± 0.12 SD vs. 10 mg CPF/kg: 0.94 ± 0.06 SD), (Fig 5B). However, there was a significant 9% increase in the MN soma diameter (ANOVA p = 0.05) identified 2 months after the last CPF exposure (0 mg CPF/kg: 1.00 ± 0.05 SD vs. 5 mg CPF/kg: 1.09 ± 0.05 SD vs. 10 mg CPF/kg: 1.09 ± 0.09 SD), (Fig 5B).

MN electrophysiological properties exist on a continuum that correspond with soma size. Overall lumbar MN soma sizes tend to distribute in a bimodal fashion where the smaller mode consists primarily of MNs that contribute to slow-type motor units and the large mode is composed primarily of the MNs of fast-type motor units. Our data also demonstrates this bimodal distribution (Fig 5C and 5D). Approximately three days following the last repeated CPF exposure, the MNs in the highest exposure group (10 mg CPF/kg per day) appear to partially dedifferentiate from the bimodal distribution with the smallest mode shifting right and largest mode shifting left (Fig 5C). The bimodal distribution is restored two months following the last CPF exposure, however it appears that only the largest mode is shifted to the right (Fig 5D).

## Discussion

OPs are neurotoxicants and include a number of commercially available pesticides and insecticides. OPs are known to disrupt neurological health and performance in humans and animals with both acute and delayed presentations (as reviewed in [17,18,41]). Acute exposures to OPs are known to inhibit ChE activity, allowing the neurotransmitter acetylcholine to accumulate at cholinergic synapses, which can lead to muscle paralysis. OPs are also capable of producing several delayed neurological conditions that appear unrelated to ChE effects. For example, survivors of acute OP toxicity often develop a neurodegenerative and paralytic disorder known as OP-Induced Delayed Neuropathy that presents as motor neuropathy weeks to months following the OP poisoning [23]. Only a few studies have examined long-term/delayed effects following repeated occupational-like CPF exposures [42,43]. These studies have indicated that cognitive effects of CPF are often delayed, detectable at least 50 days after the last repeated exposure [42,43]. Here, we show both immediate and delayed effects following two occupational weeks of oral exposure to CPF to offer further support that longer-term effects from repeated low-level or occupational-like exposures to OPs do exist.

Humans, rats, and mice express several blood esterases including AChE, butyrylcholinesterase, carboxylesterase and paraoxonase [44]. The levels of expression of each of these esterases varies to produce species-specific profiles. AChE is the largest proportion of blood esterases in human while carboxylesterase is the largest proportion of blood esterases in rats and mice [44]. A commercially available AChE kit was used to monitor blood ChE activity in this study and is also commonly used as a dosimetry for measuring OP exposure effects across many rodent studies. It has also been shown that blood AChE activity is a valid biomarker for brain AChE activity in rats exposed to CPF [45]. Although these kits are designed to specifically detect AChE, they may be detecting other cholinesterase activities as well. Multiple studies report evidence that repeated low-level, or occupational-like, OP exposures that do not severely impact AChE plasma activity levels (<15%) are linked to adverse neurological effects [12–14]. Several studies, with similar CPF exposure concentrations used herein, have reported little (<10%) to no change in AChE activity levels [12,46–49]. Multiple studies have also reported non-AChE mechanisms of CPF toxicity following low-level CPF exposures *in vivo* (<40 mg/kg) and *in vitro* (< 10 nM) [48,50–52]. Is this study, the CPF exposure concentrations used were in a lower range (<10 mg/kg) and did not produce overt signs of acute cholinergic toxicity in the rats despite a surprising 28% decrease in plasma ChE activity levels. The difference in our response in ChE activity compared to other studies is likely due to different routes of exposure, tissues sampled, and/or time courses through which the ChE activity levels were quantified. Indeed, it is unknown if the effects reported here are due to a moderate transient decrease in ChE activity levels or some other underpinning mechanism. Therefore, the causative link underpinning pathological changes following occupational CPF exposure and/or disease onset remains unclear.

When CPF is administered orally, it likely undergoes First pass metabolism in the rat liver and small intestines before reaching the blood resulting in decreased CPF blood concentration compared to other routes of exposure. In the liver, CPF is metabolized by CYP450 into chlorpyrifos-oxon (CPF-oxon), the active neurotoxic metabolite that acts as a potent AChE and butyrylcholinesterase inhibitor [34,53]. CPF-oxon can be metabolized further into several metabolites such as 3,5,6-trichloro-2-pyridinol (TCPγ), diethylphosphate, and diethylphosphorothionate. CPF-oxon inhibits human butyrylcholinesterase at a lower concentration (nM range) than AChE (μM range) and the rate of human butyrylcholinesterase inhibition by CPF-oxone is 2–3 orders of magnitude faster than human AChE inhibition [34]. Thus, it is plausible that the sub-acute exposure to low levels of CPF, performed herein, may have inhibited butyrylcholinesterase in rat plasma instead of AChE in the “immediate group” of rats analyzed 3 days post repeated exposures.

The blood or tissue concentrations of CPF and/or its metabolites were not quantified in this study. However, another study used physiologically based pharmacokinetic and pharmacodynamic (PBPK/PD) model simulations reported the daily concentrations in blood and tissue from rats exposed to 0, 3, or 10 mg/kg/day CPF subcutaneously over a 10 day period [45]. Blood CPF concentrations from rats exposed to 10 mg/kg/day had an approximate range of 0.75–9.0 μmol/l on day 1 and approximately 7.5–10.0 μmol/l on day 10 [45]. Similarly in the brain, rats exposed to 10 mg/kg/day of CPF resulted in a range of approximately 0.05–0.5 μmol/l on day 1 to approximately 0.25–0.5 μmol/l on day 10 [45]. Ellison and colleagues also reported an approximately 60% reduction of blood AChE activity and approximately 70% reduction of AChE activity in the brain on the 10^th^ day of exposure to 10 mg/kg/day CPF. In this study, there is a 28% reduction in AChE activity in the blood following the 10^th^ day of exposure to 10 mg/kg/day. This may be related to a more delayed sampling period in this study, but also due to different pharmacokinetics of oral exposures versus the subcutaneous route used by Ellison and colleague. The oral route of exposure may saturate CPF metabolism because a bolus of CPF reaches tissues over a short period of time versus the subcutaneous route which may result in a slower and more sustained CPF release into the body that never saturates metabolism. Thus, in oral exposures there may be a more rapid and greater magnitude increase in tissue CPF concentrations that metabolizes over a longer duration of time compared to subcutaneous exposures.

### Repeated chlorpyrifos exposure affects motor function

The most commonly used method to evaluate rat behavior is the open field test [54]. The test is popular because many behavior elements can be examined including locomotion, anxiety, vertical activity (rearing), and exploration drive. Following CPF exposures, there are transient but significant effects in rat behavior in the open field. A variety of central and peripheral mechanisms can impact how an animal performs in an open field test. For example, decreases in rearing behaviors can indicate heightened anxiety as well as muscle weakness or cerebellar effects [55–58]. Anxiety can also be assessed in the open field test by examining the amount of time the rats are willing to spend in the center of the arena versus the periphery and corners. Following repeated CPF exposure, the rats did not alter their exploratory time in the center of the arena suggesting no heightened anxiety or impediments to their natural exploratory behaviors. Ribeiro and colleagues, however, showed an increase in anxiety like behaviors following repeated exposures to similar CPF concentrations, and this difference may be related to different route (subcutaneous vs. oral) or increased duration (21 vs. 10 days) of the exposures compared to the exposures performed herein [42]. The rats did demonstrate an effect on locomotion behaviors with fewer beam breaks and total distance travelled following CPF exposures. Moreover, there was a decrease in rearing behavior following exposure to CPF. This observation, combined with the observation that the rats were not avoiding the center of the arena, suggest that the decrease in rearing behavior is likely related to motor behavioral changes. Overall, it is reasonable to interpret the results found in open field test as effects on motor behavior rather than anxiety and/or risk-avoidance behaviors.

The integration of sensory and motor information is necessary for executing skilled movements and learning new skills. Sensorimotor integration involves the coordinated activity of several regions of the nervous system including, but not limited to, the cerebral cortex, thalamus, basal ganglia, cerebellum, and the spinal cord. Thus, examining sensorimotor function is important for an overall evaluation of locomotor performance. Prepulse inhibition (PPI) of acoustic startle response is a well-known and widely used surrogate to provide general information regarding sensorimotor integration and processing in both humans and rats [35,59]. PPI is the normal reduction in the amplitude of the startle response that occurs following the presentation of a lower intensity acoustic stimulus prior to the acoustic startle stimulus. Although PPI is largely mediated by cholinergic muscarinic receptors in brainstem circuitry [60–62], afferent, efferent, and neuromuscular activity all contribute to the PPI response. PPI has been shown to enhance 15 minutes following treatment with physostigmine, an AChE inhibitor [63]. Thus, it is reasonable to expect that CPF, also an AChE inhibitor, will increase PPI. However, we report no changes in PPI 3 days following the final CPF exposure and a delayed reduction in PPI after a two-month recovery. A reduction of PPI is thought to reflect dysfunction of sensorimotor gating which normally suppresses excessive behavioral responses to disruptive stimuli. It is unknown if the delayed effect on sensorimotor gating is a compensatory response to an initial enhancement of PPI or reflecting some other change in sensorimotor circuitry.

### Delayed pro-inflammatory cytokine response

In addition to inhibiting ChE activity, disturbances in release of pro-inflammatory cytokines have been described as an additional mechanism of OP toxicity [64,65]. However, for lower, repeated doses of CPF, there have been mixed reports on the effects of CPF on pro-inflammatory cytokines. Some studies have reported increases [66–69], while others have reported no changes or decreases [64,70] in pro-inflammatory cytokines. The variability of effects is likely due to different model systems in addition to different routes, concentrations, and duration of CPF exposures as well as variability in experimental timing. In this study, no changes were found in pro-inflammatory cytokines three days following repeated CPF exposure. Surprisingly, we did find significant increases in pro-inflammatory cytokines that corresponded to CPF exposure concentrations two months after exposure. While it is well-known that chronic inflammation is damaging, inflammatory cytokines can play a role in recovery. For example, in damaged skeletal muscle, transient inflammatory cytokine signaling triggers a pro-myogenic signaling cascade that facilitates the repair of damaged muscle fibers [71–73]. In addition, inflammatory cytokines promote axonal regeneration and facilitate successful reinnervation of target tissue following peripheral nerve injury [74]. Although the purpose of the delayed inflammatory cytokine elevation is unknown, it does highlight the need for additional studies to determine long-term health effects of repeated exposures to OP pesticides.

### Repeated exposure to chlorpyrifos affects motoneuron soma size

The MN and the muscle fibers that it innervates, termed the motor unit, form what is described as the “final common pathway” of central nervous system integration and processing necessary to generate movement. Substantial evidence supports that MN size varies by motor unit type with slow-type motor units containing the smallest MNs, and fast-type motor units containing the largest [75,76]. Henneman and colleagues performed seminal experiments that described the relationship between the size of MNs and electrophysiological properties [77–79]. Their experiments demonstrated that in order to execute and refine skilled movement, motor units are recruited in an orderly fashion from small to large. Their observations have been supported by several studies that confirm MN size is related to its electrical excitability and MN function [75,76,80–82]. Thus, the shift in MN size observed herein following repeated CPF exposure likely reflects a physiological change in the motor unit. It is important to note that MN properties tend to exist on a continuum and the overall classification of MNs into categories, such as fast and slow types, is controversial and often used for convenience in describing specific ranges of MN properties [83].

Peripheral nerve injuries affect intrinsic and morphological properties of spinal MNs [84–86]. Briefly, spinal MNs increase input resistance, decrease rheobase current, modulate afterhyperpolarization, and decrease in MN size. Most of these changes are temporary and return to pre-axotomy states following axonal regeneration and successful reinnervation of peripheral targets [85,87–89]. There are differential changes that occur in fast and slow type MNs, and MNs appear to dedifferentiate following axotomy [85,88,90,91]. The dedifferentiation of MN properties also returns to pre-axotomy states following successful reinnervation of peripheral targets. Approximately 3 days following repeated CPF exposure, we see evidence of a transient dedifferentiation of MN soma sizes in the higher CPF exposure group (10 mg CPF/kg*bw) consistent with the observations made following axotomy. Two months later, the biphasic distribution of MN soma sizes appears fully restored. This effect may be a result of motor axons being either physically or electrochemically disconnected from their peripheral targets. Currently no studies have been published that have examined the effect of repeated low concentration exposures to CPF on neuromuscular junction structure and function.

It is unknown if the MN enlargement contributes to pathology or is a result of compensatory mechanisms to regulate MN excitability. Given that motor activity is restored at the delayed time point, it is easy to speculate that MN size plasticity may be serving a compensatory role to protect and preserve motor function. The central nervous system has the remarkable capacity to respond and adapt to environmental demands through diverse neuroplastic mechanisms. Compensatory mechanisms involve many tightly regulated intrinsic and synaptic mechanisms to maintain function and performance (as reviewed in [92–94]). Although still controversial, it has been argued that an overload of compensatory burden may eventually drive neurodegenerative pathology [95,96]. Others, however, have argued that a collapse of compensatory mechanisms lead to the development of neurodegenerative disease [97,98]. Following repeated CPF exposures, there is evidence of MN hypertrophy that is similar to what has been described in the compensatory prodromal phase in MN disease models. However, without knowing the effects on MN electrical properties and/or motor circuits, we cannot determine conclusively if these observations are compensatory or pathological in nature. Unlike the MN disease models, the environmental stimulus used to generate these effects can be removed to study the long-term outcomes of these presumed compensatory changes and recovery. It is also important to consider what vulnerabilities, such as additional stressors or genetic backgrounds, may be present during this compensatory period and if they elevate risks for permanent functional changes or disease onset.

## Conclusion

Here we show evidence of multiple effects following repeated CPF exposure with some of these effects presenting immediately, albeit transient, and others with a delayed presentation. Specifically, we show in a rat model, that repeated CPF exposure is associated with A) a mild and transient suppression of plasma ChE activity, B) transient effects in open-field motor activity, C) a delayed effect in sensorimotor integration, D) a delayed increase in systemic pro-inflammatory cytokines, E) a transient dedifferentiation of spinal lumbar MN sizes, and F) a delayed enlargement of MN somas. Some of these effects were present two months after the CPF exposures ended, highlighting the need to investigate more longer-term effects of low-level or occupational-like OP exposures. Altogether, these results offer further support that repeated occupational-like CPF exposure impacts motor function and mechanisms of the central nervous system.

## Supporting information

S1 Table Plasma AChE activity data (nmol/min/ml). (DOCX)

S2 Table Open field motor activity raw data. (DOCX)

S3 Table Acoustic startle response raw data. (DOCX)

S4 Table Pro-inflammatory cytokine mesoscale delivery data. (DOCX)

S5 Table Average lumbar motoneuron number per rat. (DOCX)

S6 Table Average lumbar motoneuron mean diameter per rat. (DOCX)

S7 Table Lumbar motoneuron diameters. (DOCX)
