# Supplementary material for: Changes in motor behavior and lumbar motoneuron morphology following repeated chlorpyrifos exposure in rats
Source: PLoS One. 2024 Jun 14;19(6):e0305173. doi: 10.1371/journal.pone.0305173 (PMC11178230; doi:10.1371/journal.pone.0305173)
Supplement: S1 Table — (DOCX) [file pone.0305173.s001.docx]

| **Supplemental Table 1. Plasma AChE Activity Data (nmol/min/ml)** | | | | | |
| --- | --- | --- | --- | --- | --- |
| Immediate Timepoint | | | Delayed Timepoint | | |
| 0 mg/kg CPF | 5 mg/kg CPF | 10 mg/kg CPF | 0 mg/kg CPF | 5 mg/kg CPF | 10 mg/kg CPF |
| 0.735616 | 0.436986 | 0.507534 | 0.829238 | 0.390663 | 0.406634 |
| 0.823288 | 0.445205 | 0.367123 | 0.694521 | 0.136364 | 0.628993 |
| 0.743836 | 0.750685 | 0.280822 | 0.234644 | 0.689041 | 0.735872 |
| 0.637671 | 0.597945 | 0.478767 | 0.371007 | 0.592138 | 0.707617 |
| 0.840411 | 0.336986 | 0.387671 | 0.786241 | 0.448403 | 0.707617 |
| 0.689726 | 0.313699 | 0.44863 | 0.716216 | 0.589681 | 0.59828 |
| 0.609589 | 0.360274 | 0.729452 | 0.685504 | 0.445946 | 0.479115 |
| 0.413014 | 0.385616 | 0.244521 | 0.643735 | 0.568796 | 0.761644 |
| 0.74863 | 0.688356 | 0.606849 | 0.331695 | 0.412776 | 0.800685 |
| 0.935616 | 0.315068 | 0.326712 | 0.34398 | 0.581081 | 0.625342 |
| 0.665231 | 0.486545 | 0.530822 | 0.905405 | 0.375921 | 0.813699 |
| 0.482239 | 0.508073 | 0.260442 | 0.379607 | 0.628993 | 0.921918 |
| 0.174381 | 0.482239 | 0.117936 | 0.588805 | 0.434876 | 0.26803 |
| 0.573735 | 0.678149 | 0.615753 | 0.351991 | 0.74704 | 0.484392 |
| 0.634247 | 0.506143 | 0.567808 | 0.613563 | 0.398278 | 0.416577 |
| 0.29817 | 0.416462 | 0.706388 | 0.504844 | 0.442411 | 0.346609 |
|  |  |  | 0.522067 |  | 0.110872 |
|  |  |  | 0.476857 |  | 0.136706 |
