# Supplementary material for: Changes in motor behavior and lumbar motoneuron morphology following repeated chlorpyrifos exposure in rats
Source: PLoS One. 2024 Jun 14;19(6):e0305173. doi: 10.1371/journal.pone.0305173 (PMC11178230; doi:10.1371/journal.pone.0305173)
Supplement: S2 Table — (DOCX) [file pone.0305173.s002.docx]

| **Supplemental Table 2. Open Field Motor Activity Raw Data** | | | | | |
| --- | --- | --- | --- | --- | --- |
| Immediate Timepoint | | | Delayed Timepoint | | |
| 0 mg/kg CPF | 5 mg/kg CPF | 10 mg/kg CPF | 0 mg/kg CPF | 5 mg/kg CPF | 10 mg/kg CPF |
| Total Ambulation/Locomotion (# Beam Breaks) | | | | | |
| 4587 | 2525 | 3965 | 3420 | 2737 | 3551 |
| 5877 | 3846 | 4013 | 4221 | 3701 | 3420 |
| 5285 | 4041 | 2568 | 5059 | 5704 | 2568 |
| 5410 | 4373 | 4584 | 5769 | 6275 | 3659 |
| 3260 | 3544 | 6129 | 2924 | 4581 | 7624 |
| 7473 | 3614 | 3455 | 7733 | 4426 | 4006 |
| 5190 | 4140 | 3170 | 4983 | 3885 | 5429 |
| 6812 | 5525 | 3559 | 5528 | 6001 | 4382 |
| 4429 | 5950 | 5197 | 2901 | 6919 | 5816 |
| 6228 | 4016 | 3362 | 4878 | 6262 | 3707 |
| 5068 | 3429 | 5363 | 5117 | 3740 | 5083 |
| 5857 | 4794 | 5519 | 5164 | 7360 | 6244 |
| Fine Movements (# Stereotypical Beam Breaks) | | | | | |
| 228 | 239 | 238 | 211 | 272 | 202 |
| 235 | 197 | 292 | 180 | 201 | 240 |
| 251 | 259 | 230 | 202 | 219 | 219 |
| 218 | 242 | 214 | 174 | 237 | 185 |
| 260 | 204 | 220 | 173 | 205 | 191 |
| 257 | 182 | 194 | 202 | 257 | 185 |
| 259 | 228 | 233 | 246 | 189 | 193 |
| 220 | 214 | 220 | 208 | 199 | 192 |
| 240 | 209 | 215 | 211 | 191 | 225 |
| 223 | 306 | 245 | 204 | 221 | 247 |
| 209 | 252 | 246 | 193 | 238 | 217 |
| 240 | 264 | 237 | 212 | 216 | 212 |
| % Time in Center | | | | | |
| 40.41353 | 38.74147 | 42.40812 | 50.44053 | 43.58034 | 58.6378 |
| 49.03632 | 38.13492 | 39.87661 | 57.79557 | 43.42883 | 44.49318 |
| 46.27291 | 52.29722 | 41.97761 | 53.58255 | 59.02174 | 53.09278 |
| 36.82023 | 39.39003 | 50.85008 | 51.71904 | 45.02239 | 48.80128 |
| 44.90374 | 40.0665 | 49.67825 | 49.93998 | 53.37506 | 56.12583 |
| 46.2591 | 38.53354 | 48.15914 | 58.77092 | 48.36783 | 47.12898 |
| 38.73362 | 40.8181 | 39.77673 | 49.24579 | 48.32041 | 42.90618 |
| 38.49522 | 47.94623 | 42.87871 | 50.20984 | 59.47199 | 43.55864 |
| 44.56029 | 51.94701 | 44.66258 | 48.88635 | 59.27393 | 49.61964 |
| 43.29359 | 39.52686 | 30.59701 | 48.21626 | 47.46228 | 39.96092 |
| 34.13521 | 35.962 | 42.41896 | 41.94456 | 45.07254 | 53.34758 |
| 36.61678 | 40.67558 | 44.76639 | 48.19422 | 50.18098 | 52.92969 |
| Total Rears | | | | | |
| 158 | 60 | 96 | 90 | 66 | 91 |
| 147 | 93 | 278 | 139 | 100 | 105 |
| 126 | 117 | 108 | 95 | 163 | 77 |
| 155 | 296 | 126 | 193 | 134 | 100 |
| 89 | 82 | 168 | 83 | 131 | 184 |
| 253 | 96 | 63 | 155 | 107 | 84 |
| 127 | 101 | 80 | 92 | 90 | 143 |
| 153 | 129 | 280 | 112 | 119 | 147 |
| 90 | 145 | 138 | 36 | 193 | 183 |
| 163 | 383 | 98 | 114 | 131 | 108 |
| 140 | 94 | 121 | 132 | 75 | 102 |
| 372 | 144 | 102 | 105 | 214 | 139 |
| Speed (cm/sec) | | | | | |
| 34.262 | 32.09 | 34.495 | 33.693 | 35.617 | 40.727 |
| 36.523 | 33.487 | 35.097 | 42.215 | 35.747 | 30.367 |
| 35.256 | 36.556 | 32.769 | 42.863 | 44.379 | 30.871 |
| 34.668 | 35.821 | 32.407 | 41.078 | 43.683 | 41.688 |
| 33.859 | 34.624 | 36.284 | 35.998 | 38.976 | 44.544 |
| 40.876 | 30.658 | 36.217 | 42.046 | 37.588 | 43.217 |
| 37.016 | 34.276 | 33.968 | 40.952 | 39.184 | 42.332 |
| 35.724 | 37.163 | 35.522 | 45.021 | 45.826 | 40.745 |
| 35.412 | 39.07 | 36.421 | 38.321 | 44.087 | 41.345 |
| 38.67 | 36.629 | 29.439 | 39.785 | 45.249 | 37.734 |
| 34.143 | 33.111 | 35.192 | 38.567 | 39.38 | 40.6 |
| 40.469 | 32.848 | 38.873 | 42.848 | 42.561 | 45.76 |
| Active Time (Sec) | | | | | |
| 1289.6 | 761.5 | 1060.8 | 1083.7 | 912.7 | 1009.4 |
| 1405.6 | 1025.8 | 1265 | 973.2 | 1100 | 1010.5 |
| 1458.3 | 1170.4 | 1464.6 | 1226.6 | 1398.2 | 1248.3 |
| 1236.3 | 1199.7 | 1204.8 | 1356.3 | 1444.1 | 1029.1 |
| 1082.4 | 961.2 | 1333 | 857.3 | 1193.7 | 1399.9 |
| 1613.5 | 931.9 | 1060.5 | 1551 | 1103.8 | 1112.8 |
| 1310.9 | 1237.1 | 1047.6 | 1235.7 | 979.1 | 1388 |
| 1580.5 | 1350.7 | 1107 | 1318.5 | 1366.8 | 1229.8 |
| 1188.2 | 1411.3 | 1359.9 | 934.9 | 1541.6 | 1448.2 |
| 1458.4 | 1203 | 1050.8 | 1185.8 | 1404.5 | 1094.7 |
| 1313.9 | 1050.4 | 1349.1 | 1241.4 | 1006.2 | 1190.8 |
| 1306.3 | 1189.6 | 1407.6 | 1266.1 | 1495.1 | 1374.7 |
| Total Distance (cm) | | | | | |
| 25876.18 | 15237.68 | 23745.39 | 23341.57 | 19386.99 | 23684.82 |
| 33276.55 | 22801.21 | 24830.38 | 26059.22 | 24769.08 | 20580.64 |
| 31357.29 | 24510.28 | 29384.04 | 31931.05 | 37957.71 | 21196.87 |
| 28533.95 | 26376.48 | 25502.13 | 37384.29 | 41055.06 | 25823.19 |
| 19848.82 | 20254.2 | 32879.28 | 19055.88 | 29630.78 | 46650.86 |
| 44555.32 | 19551.08 | 22648.13 | 47016.64 | 29045.52 | 28965.04 |
| 29645.35 | 23673.42 | 19154.21 | 31846.14 | 23862.78 | 35397.88 |
| 38160.55 | 32955.48 | 22869.24 | 37708.92 | 41327.35 | 29813.47 |
| 26144.28 | 35452.31 | 30791.87 | 20197.33 | 46247.15 | 38945.95 |
| 36529.06 | 24895.56 | 18491.65 | 31140.17 | 41694.27 | 22761.86 |
| 27562.16 | 19453.91 | 31276.47 | 30519.86 | 24560.19 | 33001.26 |
| 35117.54 | 26131.9 | 34969.04 | 34239.91 | 43898.78 | 41820.76 |
| Each value recorded per animal. | | | | | |
