# Supplementary material for: Changes in motor behavior and lumbar motoneuron morphology following repeated chlorpyrifos exposure in rats
Source: PLoS One. 2024 Jun 14;19(6):e0305173. doi: 10.1371/journal.pone.0305173 (PMC11178230; doi:10.1371/journal.pone.0305173)
Supplement: S3 Table — (DOCX) [file pone.0305173.s003.docx]

| **Supplemental Table 3. Acoustic Startle Response Raw Data** | | | | | |
| --- | --- | --- | --- | --- | --- |
| Immediate Timepoint | | | Delayed Timepoint | | |
| 0 mg/kg CPF | 5 mg/kg CPF | 10 mg/kg CPF | 0 mg/kg CPF | 5 mg/kg CPF | 10 mg/kg CPF |
| Acoustic Startle Response (Amplitude) | | | | | |
| 242.5 | 343.0833 | 178.375 | 182.2917 | 662.375 | 122.9583 |
| 316.7083 | 103.5833 | 481.9583 | 191.875 | 83.79167 | 232.625 |
| 201.875 | 308.875 | 243.1667 | 175.9167 | 170.7083 | 119.375 |
| 222.9167 | 228.9583 | 302.125 | 85.125 | 273.375 | 266.9583 |
| 357.0833 | 302.5 | 172.2917 | 295.5 | 142.375 | 88.08333 |
| 292.75 | 222.3333 | 246.6667 | 473.875 | 102.3333 | 128.75 |
| 219.5417 | 323.625 | 229.5 | 95.04167 | 383.9583 | 83.83333 |
| 209.9167 | 197 | 107.0833 | 86.16667 | 128.0417 | 158.4583 |
| 127.0417 | 269.3333 | 168.3333 | 107.6667 | 87.08333 | 149.75 |
| 289.625 | 155.0417 | 212.3333 | 143.4167 | 67 | 59.41667 |
| 83.66667 | 213.0833 | 199.7083 | 121.5417 | 154.375 | 96.16667 |
| 166.8333 | 105.875 |  | 49.45833 | 124.25 | 66.29167 |
| Prepulse Inhibition 75 dB SPL Response | | | | | |
| 49.50172 | 24.07093 | 52.97828 | 30.12571 | 73.56105 | 55.13385 |
| 46.67807 | 74.73854 | 38.1257 | 76.00434 | 77.07608 | 31.16604 |
| 63.40557 | 63.51005 | 48.78341 | 76.50403 | 62.60679 | 47.57417 |
| 63.60748 | 34.77707 | 68.032 | 75.52619 | 54.88493 | 63.1809 |
| 51.76196 | 41.25344 | 32.18863 | 56.85279 | 65.29119 | 73.50993 |
| 50.37005 | 53.35457 | 44.40878 | 64.21349 | 73.12704 | 58.73786 |
| 58.32226 | 26.98597 | 53.50399 | 67.33889 | 47.91101 | 59.29423 |
| 74.97023 | 50.46531 | 63.65759 | 65.5706 | 56.2317 | 69.52406 |
| 70.58052 | 61.71101 | 52.64851 | 70.00774 | 71.05263 | 66.94491 |
| 46.33866 | 42.8917 | 49.1562 | 82.45206 | 47.01493 | 24.3338 |
| 62.6494 | 56.72663 | 77.17505 | 76.51697 | 66.42375 | 63.56153 |
| 62.81219 | 65.28926 |  | 81.46588 | 75.62039 | 52.60842 |
| Prepulse Inhibition 85 dB SPL Response | | | | | |
| 72.74914 | 46.47802 | 80.16819 | 52.32 | 79.39863 | 73.83938 |
| 75.41113 | 87.53017 | 67.07876 | 87.557 | 86.52412 | 65.34121 |
| 81.42415 | 80.11601 | 64.73612 | 89.79157 | 73.63925 | 66.84119 |
| 78.26168 | 52.48408 | 79.09254 | 76.06461 | 74.21125 | 72.42079 |
| 73.86231 | 74.43526 | 54.29262 | 81.06317 | 77.84606 | 79.42289 |
| 69.15742 | 76.40555 | 73.98649 | 80.93731 | 89.29153 | 77.24919 |
| 77.43405 | 51.11369 | 77.95933 | 77.50986 | 62.04015 | 67.99205 |
| 82.81064 | 65.29188 | 75.83658 | 80.46422 | 74.48747 | 75.04602 |
| 83.50279 | 83.55507 | 63.24257 | 77.20588 | 76.84211 | 85.00278 |
| 78.03194 | 47.64848 | 77.74725 | 92.76583 | 72.63682 | 53.64656 |
| 74.50199 | 65.56512 | 89.06739 | 81.83065 | 85.45209 | 73.91681 |
| 79.34565 | 81.26722 |  | 78.26453 | 85.41247 | 62.60214 |
