# Supplementary material for: Changes in motor behavior and lumbar motoneuron morphology following repeated chlorpyrifos exposure in rats
Source: PLoS One. 2024 Jun 14;19(6):e0305173. doi: 10.1371/journal.pone.0305173 (PMC11178230; doi:10.1371/journal.pone.0305173)
Supplement: S4 Table — (DOCX) [file pone.0305173.s004.docx]

| **Supplemental Table 4. Pro-Inflammatory Cytokine Mesoscale Delivery Data** | | | | | |
| --- | --- | --- | --- | --- | --- |
| Immediate Timepoint | | | Delayed Timepoint | | |
| 0 mg/kg CPF | 5 mg/kg CPF | 10 mg/kg CPF | 0 mg/kg CPF | 5 mg/kg CPF | 10 mg/kg CPF |
| IFNγ | | | | | |
| 27.14483 | 27.42052 | 27.42035 | 25.21378 | 36.66291 | 41.91222 |
| 26.59227 | 26.67419 | 26.70292 | 21.98 | 35.28754 | 39.67471 |
| 26.23348 | 29.84392 | 28.4396 | 27.14636 | 38.99855 | 45.22433 |
| 30.91451 | 30.28142 | 25.57062 | 28.20221 | 40.94861 | 44.2028 |
| 23.59026 | 28.68826 | 32.91492 | 27.71318 | 39.02612 | 46.8358 |
| 30.64073 | 26.12214 | 29.76097 | 29.03035 | 36.32565 | 49.49889 |
| 28.02727 | 28.9362 | 32.04075 | 35.88413 | 40.30139 | 47.04379 |
| 27.87818 | 31.71176 | 29.62412 | 36.14375 | 43.16322 | 46.02692 |
| 35.05117 | 33.16307 | 29.34875 | 37.18089 | 38.03848 | 46.54835 |
| 34.28671 | 27.83437 | 34.34183 | 33.94016 | 39.6252 | 43.99365 |
|  |  |  | 38.19337 | 43.02998 | 44.15198 |
|  |  |  | 34.25192 | 38.71412 | 42.4904 |
| IL-10 | | | | | |
| 27.14483 | 27.42052 | 27.42035 | 25.21378 | 36.66291 | 41.91222 |
| 26.59227 | 26.67419 | 26.70292 | 21.98 | 35.28754 | 39.67471 |
| 26.23348 | 29.84392 | 28.4396 | 27.14636 | 38.99855 | 45.22433 |
| 30.91451 | 30.28142 | 25.57062 | 28.20221 | 40.94861 | 44.2028 |
| 23.59026 | 28.68826 | 32.91492 | 27.71318 | 39.02612 | 46.8358 |
| 30.64073 | 26.12214 | 29.76097 | 29.03035 | 36.32565 | 49.49889 |
| 28.02727 | 28.9362 | 32.04075 | 35.88413 | 40.30139 | 47.04379 |
| 27.87818 | 31.71176 | 29.62412 | 36.14375 | 43.16322 | 46.02692 |
| 35.05117 | 33.16307 | 29.34875 | 37.18089 | 38.03848 | 46.54835 |
| 34.28671 | 27.83437 | 34.34183 | 33.94016 | 39.6252 | 43.99365 |
|  |  |  | 38.19337 | 43.02998 | 44.15198 |
|  |  |  | 34.25192 | 38.71412 | 42.4904 |
| IL-13 | | | | | |
| 12.30835 | 11.21397 | 11.21466 | 5.968426 | 8.689982 | 9.707291 |
| 9.99132 | 12.12301 | 9.82155 | 5.757214 | 9.620635 | 10.8696 |
| 10.40601 | 10.10539 | 11.73572 | 6.491565 | 10.17973 | 9.667605 |
| 13.09039 | 12.33631 | 10.30777 | 9.466287 | 10.15605 | 10.30786 |
| 9.956848 | 11.80185 | 13.07992 | 7.894212 | 10.07633 | 11.3598 |
| 10.44518 | 10.24101 | 11.29079 | 7.24936 | 10.16209 | 11.62484 |
| 9.935538 | 10.9816 | 9.630502 | 7.288116 | 9.940602 | 10.96645 |
| 11.95082 | 11.95451 | 11.04123 | 8.266871 | 11.43804 | 10.53132 |
| 12.43569 | 13.31897 | 11.33038 | 8.919386 | 8.172898 | 8.681685 |
| 11.53279 | 8.649104 | 13.84361 | 9.773849 | 10.51061 | 9.842702 |
|  |  |  | 8.894754 | 10.48011 | 9.725567 |
|  |  |  | 10.08158 | 9.323261 | 10.73594 |
| IL-4 | | | | | |
| 2.983361 | 3.325537 | 3.264918 | 2.788235 | 3.804346 | 4.471942 |
| 3.466637 | 3.287675 | 3.392894 | 2.759022 | 3.701816 | 3.951457 |
| 3.419585 | 3.553844 | 3.800513 | 2.88477 | 3.922557 | 4.409561 |
| 3.899794 | 3.50029 | 3.399402 | 3.48278 | 4.303095 | 4.631711 |
| 2.706794 | 3.640572 | 4.018956 | 3.216608 | 3.815538 | 5.198672 |
| 3.587272 | 3.219197 | 3.893233 | 3.409151 | 3.723942 | 4.704758 |
| 3.45335 | 3.607281 | 3.77392 | 3.536488 | 4.022744 | 5.032867 |
| 3.778728 | 3.880145 | 3.694051 | 3.567858 | 4.51631 | 4.784722 |
| 4.367212 | 4.233169 | 3.677392 | 3.719666 | 4.196281 | 4.948703 |
| 4.121267 | 3.70737 | 4.285561 | 3.735275 | 4.180521 | 4.445293 |
|  |  |  | 3.860084 | 4.422733 | 4.804546 |
|  |  |  | 3.826712 | 3.891354 | 5.138628 |
| IL-6 | | | | | |
| 528.3173 | 323.8336 | 339.899 | 266.0402 | 633.9726 | 651.2175 |
| 328.6678 | 463.0578 | 346.2459 | 359.9717 | 640.5778 | 756.0593 |
| 395.9958 | 349.5419 | 411.9983 | 541.3653 | 665.7971 | 721.5028 |
| 520.3812 | 419.9602 | 296.492 | 486.1018 | 696.2689 | 681.6854 |
| 400.8077 | 453.5212 | 685.2651 | 509.7026 | 637.9148 | 821.2623 |
| 475.8287 | 322.2347 | 395.9696 | 357.3159 | 684.3359 | 984.1079 |
| 294.8838 | 283.5798 | 323.7755 | 488.6156 | 886.5868 | 838.5798 |
| 427.9797 | 491.7698 | 451.9007 | 533.4455 | 565.1414 | 648.5312 |
| 545.8034 | 585.5266 | 455.0861 | 581.0194 | 915.9665 | 619.5613 |
| 496.5149 | 310.9814 | 652.0641 | 470.187 | 758.7212 | 680.3492 |
|  |  |  | 567.7982 | 627.4135 | 694.9469 |
|  |  |  |  |  | 816.0235 |
| TNFα | | | | | |
| 4.136558 | 3.53179 | 3.185467 | 2.065828 | 2.727033 | 2.528268 |
| 2.998929 | 4.735434 | 4.430003 | 1.936515 | 3.159879 | 3.20786 |
| 5.170043 | 7.180395 | 3.915563 | 1.720022 | 2.544787 | 3.431234 |
| 4.986428 | 4.016272 | 4.933696 | 2.469165 | 2.281283 | 1.822967 |
| 3.483128 | 4.682453 | 3.618824 | 2.673487 | 1.925314 | 3.659842 |
| 4.880884 | 3.966237 | 4.720568 | 2.141262 | 3.122477 | 3.590919 |
| 3.470516 | 3.09688 | 10.22222 | 2.774966 | 2.410636 | 2.86603 |
| 3.867307 | 4.728781 | 4.202795 | 2.119967 | 2.764405 | 2.582375 |
| 4.241865 | 4.22927 | 3.828445 | 2.222383 | 3.362368 | 2.329972 |
| 3.659075 | 4.002362 | 4.089324 | 3.601298 | 2.512649 | 3.483983 |
|  |  |  | 2.270521 |  | 4.724091 |
|  |  |  | 2.367523 |  | 3.431345 |
| KC/GRO | | | | | |
| 485.5722 | 98.88117 | 148.4012 | 92.60091 | 103.2376 | 266.6642 |
| 192.2669 | 76.53401 | 153.5499 | 198.7119 | 249.3921 | 70.26942 |
| 96.72389 | 171.2652 | 304.1272 | 221.5983 | 109.0876 | 152.8817 |
| 145.5926 | 208.0221 | 301.7586 | 155.3291 | 87.25693 | 200.0915 |
| 212.3931 | 210.2432 | 87.23069 | 96.37438 | 165.5906 | 64.38066 |
| 137.7565 | 219.968 | 193.1148 | 194.8661 | 72.28108 | 176.68 |
| 180.718 | 133.7658 | 283.4656 | 175.2295 | 160.031 | 183.9875 |
| 221.7076 | 136.192 | 230.5285 | 128.0687 | 109.4857 | 77.5915 |
| 126.4496 | 239.7588 | 117.714 | 127.3585 | 118.2154 | 316.7317 |
| 288.1605 | 334.3594 | 119.7764 | 139.4887 | 166.2938 | 182.5445 |
|  |  |  | 104.6611 | 209.9345 | 155.4818 |
|  |  |  | 130.9387 | 89.78973 | 168.2045 |
