# Supplementary material for: Changes in motor behavior and lumbar motoneuron morphology following repeated chlorpyrifos exposure in rats
Source: PLoS One. 2024 Jun 14;19(6):e0305173. doi: 10.1371/journal.pone.0305173 (PMC11178230; doi:10.1371/journal.pone.0305173)
Supplement: S5 Table — (DOCX) [file pone.0305173.s005.docx]

| **Supplemental Table 5. Average Lumbar Motoneuron Number per Rat.** | | | | | |
| --- | --- | --- | --- | --- | --- |
| Immediate Timepoint | | | Delayed Timepoint | | |
| 0 mg/kg CPF | 5 mg/kg CPF | 10 mg/kg CPF | 0 mg/kg CPF | 5 mg/kg CPF | 10 mg/kg CPF |
| 26.83333 | 13.66667 | 19.16667 | 10.16667 | 16 | 14.66667 |
| 20.83333 | 4.5 | 7.166667 | 17.5 | 22.5 | 20.66667 |
| 14.66667 | 19.16667 | 28.83333 | 19 | 15.66667 | 25.16667 |
| 13 | 22 | 15.66667 | 12.16667 | 30.66667 | 18.5 |
| 15.66667 |  | 18.16667 | 27.16667 | 28.5 | 25 |
| 11.83333 |  | 12.66667 | 30.5 | 26.33333 | 31.16667 |
| Motoneurons were quantified unilaterally in lamina IX of 70 µm think spinal cord slices. | | | | | |
