# Supplementary material for: Changes in motor behavior and lumbar motoneuron morphology following repeated chlorpyrifos exposure in rats
Source: PLoS One. 2024 Jun 14;19(6):e0305173. doi: 10.1371/journal.pone.0305173 (PMC11178230; doi:10.1371/journal.pone.0305173)
Supplement: S6 Table — (DOCX) [file pone.0305173.s006.docx]

| **Supplemental Table 6. Average Lumbar Motoneuron Mean Diameter per Rat.** | | | | | |
| --- | --- | --- | --- | --- | --- |
| Immediate Timepoint | | | Delayed Timepoint | | |
| 0 mg/kg CPF | 5 mg/kg CPF | 10 mg/kg CPF | 0 mg/kg CPF | 5 mg/kg CPF | 10 mg/kg CPF |
| 21.1719 | 21.7763 | 20.70341 | 18.58019 | 21.85687 | 22.37841 |
| 21.32114 | 15.91108 | 18.60023 | 18.37823 | 21.35501 | 18.49493 |
| 20.52917 | 20.13894 | 21.25091 | 17.41861 | 18.33142 | 19.05239 |
| 21.29386 | 20.2971 | 20.19339 | 19.14044 | 20.20455 | 20.37948 |
| 20.51395 |  | 20.19969 | 19.86973 | 20.75926 | 21.88042 |
| 21.85199 |  | 17.96484 | 20.15247 | 20.24979 | 21.92842 |
| Motoneurons mean diameters were measured in single optical sections through the nucleolus. | | | | | |
