# Supplementary material for: Changes in motor behavior and lumbar motoneuron morphology following repeated chlorpyrifos exposure in rats
Source: PLoS One. 2024 Jun 14;19(6):e0305173. doi: 10.1371/journal.pone.0305173 (PMC11178230; doi:10.1371/journal.pone.0305173)
Supplement: S7 Table — (DOCX) [file pone.0305173.s007.docx]

| **Supplemental Table 7. Lumbar Motoneuron Diameters (µm).** | | | | | |
| --- | --- | --- | --- | --- | --- |
| Immediate Timepoint | | | Delayed Timepoint | | |
| 0 mg/kg CPF | 5 mg/kg CPF | 10 mg/kg CPF | 0 mg/kg CPF | 5 mg/kg CPF | 10 mg/kg CPF |
| 16.05236 | 16.14317 | 16.05603 | 16.04512 | 16.00328 | 16.03926 |
| 16.12807 | 16.22356 | 16.155 | 16.05564 | 16.00856 | 16.04661 |
| 16.17598 | 16.28381 | 16.24298 | 16.07041 | 16.12333 | 16.06961 |
| 16.20087 | 16.395 | 16.25298 | 16.11138 | 16.18346 | 16.09644 |
| 16.20814 | 16.53186 | 16.38267 | 16.11296 | 16.19055 | 16.10416 |
| 16.50285 | 16.55093 | 16.42208 | 16.20166 | 16.19241 | 16.13626 |
| 16.62446 | 16.59886 | 16.44166 | 16.20952 | 16.19694 | 16.22709 |
| 16.77644 | 16.76343 | 16.47223 | 16.21374 | 16.34013 | 16.25866 |
| 16.79086 | 16.91455 | 16.4761 | 16.22042 | 16.41907 | 16.26767 |
| 16.90334 | 16.93684 | 16.48315 | 16.24994 | 16.42974 | 16.35212 |
| 16.91003 | 17.06581 | 16.48499 | 16.32717 | 16.43061 | 16.40481 |
| 16.9484 | 17.07757 | 16.5097 | 16.34082 | 16.55381 | 16.45772 |
| 17.07281 | 17.08633 | 16.54188 | 16.38918 | 16.55795 | 16.61056 |
| 17.15181 | 17.12636 | 16.68088 | 16.41665 | 16.5617 | 16.62973 |
| 17.17621 | 17.26249 | 16.6907 | 16.431 | 16.66913 | 16.7052 |
| 17.17797 | 17.37347 | 16.69137 | 16.46604 | 16.72873 | 16.74214 |
| 17.19567 | 17.40378 | 16.72178 | 16.4674 | 16.78821 | 16.78498 |
| 17.22315 | 17.46197 | 16.76761 | 16.49619 | 16.80944 | 16.80859 |
| 17.28508 | 17.47491 | 16.80461 | 16.59176 | 16.85221 | 16.82052 |
| 17.34651 | 17.56281 | 16.81115 | 16.62158 | 16.87581 | 16.83434 |
| 17.58935 | 17.63221 | 16.8315 | 16.65307 | 16.90918 | 16.83595 |
| 17.7293 | 17.75577 | 16.85211 | 16.69547 | 16.91125 | 16.85938 |
| 17.75577 | 17.77684 | 16.88553 | 16.697 | 16.95921 | 16.88175 |
| 17.76689 | 17.82139 | 16.90513 | 16.73101 | 17.03873 | 16.89797 |
| 17.82032 | 17.82184 | 16.91869 | 16.73377 | 17.07216 | 16.9639 |
| 17.82559 | 17.83139 | 16.93054 | 16.74528 | 17.10598 | 16.98341 |
| 17.82737 | 17.85478 | 16.93844 | 16.77872 | 17.1363 | 16.99457 |
| 17.8521 | 17.90438 | 16.94756 | 16.83926 | 17.17853 | 17.0162 |
| 17.90358 | 17.97034 | 16.99072 | 16.89071 | 17.19928 | 17.02462 |
| 17.92785 | 17.99142 | 17.01872 | 16.94465 | 17.20752 | 17.03191 |
| 18.02617 | 18.05088 | 17.07253 | 16.97469 | 17.23036 | 17.08111 |
| 18.11007 | 18.12738 | 17.20696 | 17.03565 | 17.25539 | 17.11147 |
| 18.11525 | 18.14898 | 17.214 | 17.07813 | 17.28305 | 17.11975 |
| 18.15354 | 18.18623 | 17.23701 | 17.11789 | 17.3172 | 17.15905 |
| 18.19831 | 18.25239 | 17.26351 | 17.13909 | 17.44801 | 17.19595 |
| 18.21309 | 18.32379 | 17.35889 | 17.24939 | 17.45093 | 17.2213 |
| 18.23982 | 18.35487 | 17.36962 | 17.26683 | 17.46461 | 17.25705 |
| 18.32309 | 18.3872 | 17.37237 | 17.32327 | 17.49886 | 17.28618 |
| 18.37273 | 18.47317 | 17.39537 | 17.32997 | 17.52032 | 17.29198 |
| 18.42492 | 18.47644 | 17.42637 | 17.39564 | 17.54249 | 17.29963 |
| 18.44082 | 18.5353 | 17.4271 | 17.3982 | 17.5764 | 17.30708 |
| 18.50882 | 18.56517 | 17.43021 | 17.40781 | 17.58247 | 17.31481 |
| 18.51132 | 18.58566 | 17.44984 | 17.42125 | 17.58736 | 17.37008 |
| 18.60056 | 18.64451 | 17.5687 | 17.43276 | 17.60781 | 17.39482 |
| 18.60322 | 18.69296 | 17.5764 | 17.44418 | 17.69181 | 17.39637 |
| 18.68674 | 18.71305 | 17.586 | 17.45376 | 17.69568 | 17.46726 |
| 18.75105 | 18.74001 | 17.61848 | 17.49076 | 17.72571 | 17.46853 |
| 18.76158 | 18.77956 | 17.66253 | 17.49986 | 17.77092 | 17.48648 |
| 18.80845 | 18.84787 | 17.72121 | 17.53777 | 17.7797 | 17.65901 |
| 18.91693 | 18.85758 | 17.72993 | 17.60383 | 17.81022 | 17.6655 |
| 18.92223 | 18.88289 | 17.75703 | 17.61532 | 17.84487 | 17.6955 |
| 18.9314 | 18.92602 | 17.76815 | 17.64738 | 17.87786 | 17.72598 |
| 18.95864 | 18.96838 | 17.79251 | 17.67289 | 17.88285 | 17.7433 |
| 18.98088 | 18.97341 | 17.79438 | 17.71357 | 17.92953 | 17.77173 |
| 18.98088 | 18.98994 | 17.80816 | 17.7346 | 18.03659 | 17.82523 |
| 18.99413 | 19.03977 | 17.86993 | 17.81531 | 18.03915 | 17.83175 |
| 19.00377 | 19.06091 | 17.88872 | 17.86601 | 18.04285 | 17.84059 |
| 19.05323 | 19.0615 | 17.98956 | 17.86824 | 18.05812 | 17.84407 |
| 19.07327 | 19.08012 | 17.99487 | 17.93664 | 18.07935 | 17.85326 |
| 19.07336 | 19.11139 | 17.99903 | 17.94028 | 18.0974 | 17.87055 |
| 19.07987 | 19.1486 | 18.04921 | 17.94143 | 18.10936 | 17.87376 |
| 19.08087 | 19.15691 | 18.09309 | 17.96467 | 18.11974 | 17.88133 |
| 19.08988 | 19.20597 | 18.09951 | 17.97822 | 18.12149 | 17.96352 |
| 19.12597 | 19.29052 | 18.10778 | 18.00619 | 18.1301 | 18.01406 |
| 19.12763 | 19.29894 | 18.1127 | 18.03394 | 18.14055 | 18.01742 |
| 19.14111 | 19.35628 | 18.14792 | 18.03809 | 18.14529 | 18.03562 |
| 19.14311 | 19.44165 | 18.15117 | 18.04303 | 18.1567 | 18.04418 |
| 19.15824 | 19.47177 | 18.16941 | 18.05847 | 18.19087 | 18.07169 |
| 19.17843 | 19.49245 | 18.18605 | 18.07372 | 18.22628 | 18.1127 |
| 19.18997 | 19.55215 | 18.22052 | 18.09687 | 18.2296 | 18.12852 |
| 19.23447 | 19.57096 | 18.22393 | 18.10629 | 18.25553 | 18.12905 |
| 19.23845 | 19.59697 | 18.22436 | 18.11314 | 18.29918 | 18.13204 |
| 19.25549 | 19.6038 | 18.25178 | 18.13195 | 18.31127 | 18.13616 |
| 19.25615 | 19.64234 | 18.30266 | 18.13335 | 18.31735 | 18.14125 |
| 19.30595 | 19.67256 | 18.30988 | 18.13757 | 18.32153 | 18.15512 |
| 19.35126 | 19.70006 | 18.38573 | 18.22628 | 18.37117 | 18.16283 |
| 19.3724 | 19.70951 | 18.43486 | 18.2775 | 18.37395 | 18.20837 |
| 19.37248 | 19.77807 | 18.4592 | 18.30779 | 18.40884 | 18.20977 |
| 19.47553 | 19.78483 | 18.48049 | 18.32683 | 18.42751 | 18.24096 |
| 19.52134 | 19.84101 | 18.48334 | 18.35461 | 18.439 | 18.24872 |
| 19.53977 | 19.89919 | 18.49479 | 18.36415 | 18.50891 | 18.27872 |
| 19.58933 | 19.90095 | 18.49987 | 18.36987 | 18.52163 | 18.28281 |
| 19.63642 | 19.939 | 18.50538 | 18.37299 | 18.53865 | 18.31709 |
| 19.66811 | 19.94571 | 18.5292 | 18.39629 | 18.54372 | 18.32439 |
| 19.66892 | 19.96996 | 18.53118 | 18.40425 | 18.57306 | 18.36562 |
| 19.67393 | 19.97403 | 18.53178 | 18.42743 | 18.60518 | 18.371 |
| 19.67701 | 19.9855 | 18.54303 | 18.46205 | 18.60672 | 18.41835 |
| 19.68073 | 20.0043 | 18.54346 | 18.48575 | 18.62093 | 18.43209 |
| 19.68607 | 20.01409 | 18.54646 | 18.53882 | 18.64169 | 18.45868 |
| 19.71097 | 20.06264 | 18.54715 | 18.542 | 18.64212 | 18.47188 |
| 19.71565 | 20.06415 | 18.55196 | 18.54844 | 18.64315 | 18.47558 |
| 19.72026 | 20.06986 | 18.56045 | 18.55179 | 18.64349 | 18.53075 |
| 19.7485 | 20.12786 | 18.56997 | 18.60304 | 18.64417 | 18.54045 |
| 19.75084 | 20.16571 | 18.57169 | 18.68793 | 18.65339 | 18.55771 |
| 19.75309 | 20.17834 | 18.58 | 18.70514 | 18.67276 | 18.57675 |
| 19.77171 | 20.19475 | 18.5866 | 18.72802 | 18.78541 | 18.5914 |
| 19.77533 | 20.21635 | 18.6039 | 18.79389 | 18.8143 | 18.59166 |
| 19.78813 | 20.24437 | 18.62007 | 18.79609 | 18.85429 | 18.60381 |
| 19.82808 | 20.25813 | 18.68308 | 18.80608 | 18.85741 | 18.61853 |
| 19.84117 | 20.29104 | 18.68751 | 18.85302 | 18.88922 | 18.7042 |
| 19.84775 | 20.29849 | 18.7059 | 18.85775 | 18.90304 | 18.71076 |
| 19.85962 | 20.30061 | 18.70701 | 18.90843 | 18.90818 | 18.75275 |
| 19.86611 | 20.37061 | 18.71135 | 18.92518 | 18.92627 | 18.77583 |
| 19.86619 | 20.41527 | 18.71578 | 18.94393 | 18.94091 | 18.79795 |
| 19.88045 | 20.42058 | 18.72828 | 19.0191 | 18.94267 | 18.82039 |
| 19.90535 | 20.43391 | 18.73261 | 19.02303 | 18.96082 | 18.8526 |
| 19.90719 | 20.4642 | 18.74137 | 19.0299 | 18.97778 | 18.87648 |
| 19.90903 | 20.46614 | 18.77176 | 19.04679 | 18.98491 | 18.90001 |
| 19.92118 | 20.4684 | 18.79939 | 19.04947 | 18.98608 | 18.9054 |
| 19.9259 | 20.47851 | 18.80253 | 19.05883 | 18.98818 | 18.91331 |
| 19.94714 | 20.4845 | 18.89428 | 19.07954 | 19.01977 | 18.94553 |
| 19.97506 | 20.50656 | 18.90076 | 19.08221 | 19.03358 | 18.97425 |
| 19.9761 | 20.5269 | 18.9155 | 19.08304 | 19.04562 | 19.01835 |
| 19.98638 | 20.54396 | 18.93124 | 19.08429 | 19.05047 | 19.02554 |
| 19.98694 | 20.55752 | 18.93796 | 19.11789 | 19.06451 | 19.04077 |
| 20.00724 | 20.57803 | 19.00561 | 19.18972 | 19.08421 | 19.07528 |
| 20.01504 | 20.58469 | 19.01005 | 19.20788 | 19.12305 | 19.07853 |
| 20.04485 | 20.58616 | 19.01793 | 19.21194 | 19.13471 | 19.15192 |
| 20.0466 | 20.61561 | 19.02906 | 19.23381 | 19.16564 | 19.15791 |
| 20.07153 | 20.62356 | 19.03692 | 19.25673 | 19.18897 | 19.15974 |
| 20.07304 | 20.64871 | 19.04102 | 19.27557 | 19.20382 | 19.17286 |
| 20.08636 | 20.66105 | 19.07937 | 19.27921 | 19.22305 | 19.17394 |
| 20.0984 | 20.67677 | 19.09539 | 19.37429 | 19.22387 | 19.20597 |
| 20.11274 | 20.72484 | 19.11122 | 19.3853 | 19.23729 | 19.21733 |
| 20.12168 | 20.76683 | 19.11422 | 19.38752 | 19.24614 | 19.24953 |
| 20.133 | 20.77495 | 19.13296 | 19.42739 | 19.25375 | 19.27566 |
| 20.15094 | 20.80337 | 19.13645 | 19.43624 | 19.2564 | 19.34023 |
| 20.19412 | 20.8098 | 19.14868 | 19.45753 | 19.29407 | 19.34402 |
| 20.19475 | 20.86085 | 19.17021 | 19.47128 | 19.29415 | 19.36516 |
| 20.19735 | 20.86696 | 19.23042 | 19.50167 | 19.30727 | 19.38136 |
| 20.20957 | 20.92716 | 19.23249 | 19.52868 | 19.32368 | 19.39482 |
| 20.24822 | 20.93765 | 19.2545 | 19.55818 | 19.37018 | 19.39565 |
| 20.26473 | 20.94898 | 19.29308 | 19.56884 | 19.3756 | 19.40418 |
| 20.31543 | 20.97595 | 19.29424 | 19.57218 | 19.3885 | 19.40689 |
| 20.31825 | 20.98855 | 19.30339 | 19.59218 | 19.38916 | 19.42018 |
| 20.34542 | 20.99006 | 19.34632 | 19.6176 | 19.44337 | 19.42903 |
| 20.3667 | 20.99113 | 19.36245 | 19.64648 | 19.44918 | 19.46162 |
| 20.36873 | 21.01251 | 19.36409 | 19.66786 | 19.45499 | 19.48231 |
| 20.37468 | 21.02334 | 19.36944 | 19.68 | 19.51881 | 19.49245 |
| 20.39335 | 21.03848 | 19.37051 | 19.68 | 19.54816 | 19.53952 |
| 20.39428 | 21.0515 | 19.41255 | 19.72631 | 19.55606 | 19.54946 |
| 20.40583 | 21.07033 | 19.42321 | 19.74906 | 19.56119 | 19.5528 |
| 20.41894 | 21.10204 | 19.42542 | 19.76768 | 19.56363 | 19.57079 |
| 20.4318 | 21.10634 | 19.42731 | 19.81933 | 19.58104 | 19.58283 |
| 20.4417 | 21.13899 | 19.44329 | 19.81973 | 19.58316 | 19.58348 |
| 20.45735 | 21.14441 | 19.45033 | 19.82037 | 19.60364 | 19.60258 |
| 20.45766 | 21.15322 | 19.47749 | 19.82848 | 19.61785 | 19.64696 |
| 20.46505 | 21.1703 | 19.48346 | 19.88902 | 19.62093 | 19.66098 |
| 20.49887 | 21.21508 | 19.48542 | 19.91007 | 19.64194 | 19.66576 |
| 20.50283 | 21.21561 | 19.49171 | 19.95864 | 19.65102 | 19.69133 |
| 20.50563 | 21.23684 | 19.51465 | 19.96438 | 19.66284 | 19.6923 |
| 20.5262 | 21.28836 | 19.51661 | 19.98789 | 19.68186 | 19.71549 |
| 20.54124 | 21.35274 | 19.52803 | 19.98989 | 19.69529 | 19.71913 |
| 20.54744 | 21.3637 | 19.53708 | 20.00271 | 19.73454 | 19.72284 |
| 20.56061 | 21.37845 | 19.58885 | 20.00931 | 19.73906 | 19.72413 |
| 20.56185 | 21.392 | 19.60185 | 20.01647 | 19.74326 | 19.73971 |
| 20.56317 | 21.3961 | 19.63788 | 20.06732 | 19.75704 | 19.75382 |
| 20.56774 | 21.45095 | 19.66098 | 20.09642 | 19.75777 | 19.7626 |
| 20.58275 | 21.49544 | 19.68575 | 20.09801 | 19.77324 | 19.76945 |
| 20.58755 | 21.51285 | 19.70046 | 20.09936 | 19.77356 | 19.79312 |
| 20.59196 | 21.51285 | 19.70709 | 20.09975 | 19.79441 | 19.80679 |
| 20.5925 | 21.52942 | 19.71792 | 20.12255 | 19.79827 | 19.84189 |
| 20.59466 | 21.53896 | 19.71888 | 20.14075 | 19.80108 | 19.90359 |
| 20.6363 | 21.54088 | 19.74035 | 20.18119 | 19.85938 | 19.91287 |
| 20.63892 | 21.60834 | 19.74793 | 20.22343 | 19.86635 | 19.93133 |
| 20.65457 | 21.63184 | 19.79851 | 20.22918 | 19.86956 | 19.95752 |
| 20.69124 | 21.65361 | 19.84173 | 20.23005 | 19.87413 | 19.96677 |
| 20.69639 | 21.65398 | 19.85906 | 20.2387 | 19.93764 | 19.97379 |
| 20.71147 | 21.67037 | 19.92102 | 20.24012 | 19.96079 | 19.97506 |
| 20.71362 | 21.6714 | 19.92198 | 20.25931 | 19.96845 | 19.98837 |
| 20.77886 | 21.67838 | 19.92654 | 20.28405 | 19.98025 | 20.01671 |
| 20.78208 | 21.70481 | 19.94483 | 20.29025 | 20.0047 | 20.02769 |
| 20.79265 | 21.71412 | 19.96262 | 20.29786 | 20.01011 | 20.03262 |
| 20.80467 | 21.71706 | 19.96964 | 20.29802 | 20.0121 | 20.03683 |
| 20.80896 | 21.72263 | 19.97251 | 20.30288 | 20.01767 | 20.04708 |
| 20.81095 | 21.74703 | 19.98949 | 20.31339 | 20.0624 | 20.04748 |
| 20.81401 | 21.77542 | 20.00884 | 20.31621 | 20.09127 | 20.06637 |
| 20.82517 | 21.79997 | 20.01162 | 20.33149 | 20.09191 | 20.0862 |
| 20.8306 | 21.81852 | 20.02761 | 20.33212 | 20.12754 | 20.08889 |
| 20.83626 | 21.81852 | 20.05367 | 20.34832 | 20.13268 | 20.10237 |
| 20.85001 | 21.8326 | 20.06082 | 20.35255 | 20.14928 | 20.11963 |
| 20.86024 | 21.95283 | 20.06455 | 20.35959 | 20.15663 | 20.13363 |
| 20.87291 | 21.95312 | 20.08453 | 20.36787 | 20.16192 | 20.14241 |
| 20.88321 | 21.95783 | 20.11361 | 20.40622 | 20.16295 | 20.16169 |
| 20.8893 | 22.0238 | 20.12517 | 20.4127 | 20.17906 | 20.16335 |
| 20.91125 | 22.09714 | 20.16058 | 20.42463 | 20.17984 | 20.17606 |
| 20.91795 | 22.13688 | 20.16856 | 20.42939 | 20.19396 | 20.18103 |
| 20.94609 | 22.14306 | 20.20169 | 20.4452 | 20.20043 | 20.18955 |
| 20.94822 | 22.16111 | 20.21666 | 20.44567 | 20.20303 | 20.20429 |
| 20.96965 | 22.1744 | 20.22894 | 20.45112 | 20.23209 | 20.21808 |
| 20.96972 | 22.21077 | 20.23178 | 20.46902 | 20.25522 | 20.22131 |
| 20.97496 | 22.22231 | 20.24028 | 20.47003 | 20.25891 | 20.22871 |
| 20.9764 | 22.27864 | 20.24185 | 20.4845 | 20.27298 | 20.25404 |
| 20.97914 | 22.32398 | 20.24405 | 20.56178 | 20.28695 | 20.25663 |
| 21.00652 | 22.32911 | 20.26622 | 20.57687 | 20.3293 | 20.25734 |
| 21.01592 | 22.3542 | 20.28185 | 20.58043 | 20.33149 | 20.28876 |
| 21.02985 | 22.36723 | 20.29072 | 20.59528 | 20.33376 | 20.29064 |
| 21.0431 | 22.47412 | 20.3195 | 20.61252 | 20.3394 | 20.29331 |
| 21.06549 | 22.47759 | 20.32013 | 20.61445 | 20.34073 | 20.297 |
| 21.08151 | 22.54422 | 20.34339 | 20.66352 | 20.34284 | 20.29959 |
| 21.08521 | 22.55348 | 20.3785 | 20.66529 | 20.36092 | 20.3177 |
| 21.10023 | 22.56004 | 20.38202 | 20.68755 | 20.38811 | 20.32742 |
| 21.10913 | 22.56533 | 20.38319 | 20.69062 | 20.3942 | 20.33783 |
| 21.10944 | 22.57754 | 20.39475 | 20.70547 | 20.3999 | 20.34362 |
| 21.12165 | 22.57881 | 20.41169 | 20.70563 | 20.40279 | 20.34621 |
| 21.15149 | 22.57951 | 20.41418 | 20.74704 | 20.40466 | 20.36357 |
| 21.15179 | 22.60327 | 20.42214 | 20.74926 | 20.4385 | 20.37061 |
| 21.16481 | 22.61066 | 20.44575 | 20.75862 | 20.43921 | 20.38233 |
| 21.1915 | 22.65899 | 20.45027 | 20.78415 | 20.44902 | 20.38772 |
| 21.19308 | 22.6618 | 20.45042 | 20.78944 | 20.45439 | 20.45089 |
| 21.19683 | 22.67262 | 20.4586 | 20.82517 | 20.45992 | 20.49017 |
| 21.20036 | 22.69312 | 20.46124 | 20.83695 | 20.46039 | 20.50532 |
| 21.21425 | 22.69431 | 20.46902 | 20.85398 | 20.46054 | 20.52449 |
| 21.22003 | 22.70946 | 20.47921 | 20.86879 | 20.48046 | 20.52457 |
| 21.22341 | 22.75094 | 20.50416 | 20.87703 | 20.54225 | 20.54031 |
| 21.22439 | 22.75416 | 20.50796 | 20.8903 | 20.56379 | 20.54186 |
| 21.22664 | 22.85004 | 20.51487 | 20.89342 | 20.57966 | 20.5431 |
| 21.23504 | 22.85986 | 20.51945 | 20.90866 | 20.57973 | 20.54814 |
| 21.24313 | 22.90829 | 20.5262 | 20.94206 | 20.61615 | 20.57455 |
| 21.26314 | 22.95384 | 20.53388 | 20.94336 | 20.61661 | 20.58507 |
| 21.26898 | 22.96085 | 20.55752 | 20.95141 | 20.64401 | 20.58979 |
| 21.27699 | 22.99785 | 20.56108 | 20.9676 | 20.65866 | 20.5935 |
| 21.28425 | 23.00194 | 20.57749 | 20.97291 | 20.66714 | 20.60332 |
| 21.29322 | 23.00367 | 20.59211 | 20.98521 | 20.68216 | 20.60587 |
| 21.30541 | 23.00533 | 20.59304 | 20.98597 | 20.68978 | 20.61877 |
| 21.32505 | 23.00692 | 20.59466 | 21.00432 | 20.69255 | 20.62958 |
| 21.33715 | 23.07741 | 20.59806 | 21.03652 | 20.69855 | 20.65072 |
| 21.34513 | 23.08583 | 20.6197 | 21.05853 | 20.6997 | 20.67831 |
| 21.34692 | 23.09059 | 20.65758 | 21.07085 | 20.71385 | 20.68092 |
| 21.35125 | 23.18377 | 20.65789 | 21.07199 | 20.71439 | 20.68685 |
| 21.36728 | 23.21967 | 20.65928 | 21.07735 | 20.74235 | 20.69955 |
| 21.38664 | 23.25886 | 20.67215 | 21.09699 | 20.74312 | 20.70409 |
| 21.38798 | 23.26611 | 20.67307 | 21.11426 | 20.74811 | 20.72208 |
| 21.38806 | 23.27611 | 20.68108 | 21.11653 | 20.75394 | 20.73276 |
| 21.39171 | 23.27932 | 20.70547 | 21.12896 | 20.75716 | 20.74435 |
| 21.4196 | 23.32019 | 20.71316 | 21.14094 | 20.77219 | 20.74634 |
| 21.42012 | 23.35103 | 20.72384 | 21.15743 | 20.78974 | 20.75341 |
| 21.43283 | 23.36317 | 20.72968 | 21.18413 | 20.80199 | 20.77012 |
| 21.43528 | 23.36937 | 20.73076 | 21.18654 | 20.80452 | 20.78438 |
| 21.43677 | 23.3738 | 20.75141 | 21.18879 | 20.82097 | 20.80995 |
| 21.44033 | 23.44373 | 20.78522 | 21.22116 | 20.85177 | 20.81401 |
| 21.44204 | 23.46973 | 20.80153 | 21.23624 | 20.85894 | 20.81431 |
| 21.44843 | 23.48044 | 20.82831 | 21.24246 | 20.86413 | 20.82097 |
| 21.45385 | 23.51392 | 20.8723 | 21.30399 | 20.8739 | 20.8212 |
| 21.45555 | 23.51744 | 20.89129 | 21.31467 | 20.88008 | 20.83977 |
| 21.49611 | 23.51859 | 20.9018 | 21.31818 | 20.88809 | 20.84336 |
| 21.511 | 23.52177 | 20.90569 | 21.31945 | 20.90607 | 20.85474 |
| 21.5448 | 23.53937 | 20.92206 | 21.33289 | 20.91247 | 20.91071 |
| 21.54864 | 23.58627 | 20.9299 | 21.34804 | 20.91939 | 20.91932 |
| 21.55123 | 23.61454 | 20.95309 | 21.3631 | 20.9394 | 20.92274 |
| 21.55167 | 23.6291 | 20.9764 | 21.36608 | 20.95005 | 20.93423 |
| 21.56415 | 23.63564 | 20.98931 | 21.36728 | 20.95081 | 20.94556 |
| 21.57124 | 23.66466 | 20.99219 | 21.36772 | 20.9543 | 20.9619 |
| 21.57353 | 23.68181 | 21.03084 | 21.37324 | 20.95841 | 20.96676 |
| 21.5736 | 23.75003 | 21.07055 | 21.38456 | 20.97162 | 20.97694 |
| 21.57641 | 23.7712 | 21.08362 | 21.40391 | 20.98453 | 20.9959 |
| 21.5832 | 23.85953 | 21.08513 | 21.40867 | 20.9956 | 20.99909 |
| 21.61217 | 23.906 | 21.08929 | 21.41722 | 21.0025 | 21.00129 |
| 21.61645 | 23.95863 | 21.09736 | 21.46394 | 21.00409 | 21.02546 |
| 21.62889 | 23.96401 | 21.12738 | 21.49789 | 21.00447 | 21.02819 |
| 21.63794 | 24.09971 | 21.12798 | 21.53179 | 21.04802 | 21.0431 |
| 21.64192 | 24.15489 | 21.13748 | 21.54184 | 21.05415 | 21.05883 |
| 21.66295 | 24.17545 | 21.13853 | 21.55293 | 21.05581 | 21.05974 |
| 21.67023 | 24.18006 | 21.1478 | 21.57013 | 21.0667 | 21.06934 |
| 21.67978 | 24.21559 | 21.14817 | 21.57685 | 21.06859 | 21.07697 |
| 21.70679 | 24.23787 | 21.14825 | 21.59006 | 21.07161 | 21.08747 |
| 21.71288 | 24.24707 | 21.17323 | 21.5902 | 21.08347 | 21.11381 |
| 21.71478 | 24.25048 | 21.20292 | 21.6034 | 21.10672 | 21.12158 |
| 21.75522 | 24.27234 | 21.2549 | 21.61696 | 21.13122 | 21.12369 |
| 21.75962 | 24.54386 | 21.26658 | 21.63095 | 21.13605 | 21.13288 |
| 21.77637 | 24.58205 | 21.27467 | 21.66229 | 21.1429 | 21.15736 |
| 21.77798 | 24.65352 | 21.30451 | 21.70562 | 21.15224 | 21.16744 |
| 21.78792 | 24.67928 | 21.3199 | 21.71691 | 21.16315 | 21.16879 |
| 21.79683 | 24.70263 | 21.33371 | 21.71735 | 21.17037 | 21.17075 |
| 21.80209 | 24.76958 | 21.34051 | 21.74556 | 21.18443 | 21.18819 |
| 21.81078 | 24.86979 | 21.34289 | 21.74878 | 21.18541 | 21.19901 |
| 21.8191 | 24.87357 | 21.34744 | 21.76342 | 21.18752 | 21.23279 |
| 21.84186 | 25.1405 | 21.35065 | 21.77227 | 21.20825 | 21.23849 |
| 21.84784 | 25.16247 | 21.35438 | 21.81056 | 21.23339 | 21.24621 |
| 21.85097 | 25.21658 | 21.37279 | 21.8191 | 21.23489 | 21.25497 |
| 21.85746 | 25.60852 | 21.37771 | 21.84062 | 21.24403 | 21.26464 |
| 21.86445 | 25.68718 | 21.37786 | 21.8587 | 21.25115 | 21.26905 |
| 21.90403 | 25.91332 | 21.39826 | 21.86277 | 21.26658 | 21.28963 |
| 21.91072 | 26.09952 | 21.40703 | 21.86998 | 21.26718 | 21.31856 |
| 21.91203 | 26.56431 | 21.4335 | 21.88854 | 21.27339 | 21.32379 |
| 21.92982 | 26.63859 | 21.45451 | 21.89851 | 21.27766 | 21.32789 |
| 21.93832 | 26.8464 | 21.46067 | 21.91225 | 21.28021 | 21.32983 |
| 21.9442 | 27.34248 | 21.46639 | 21.92452 | 21.29113 | 21.33207 |
| 21.95181 | 27.64153 | 21.47061 | 21.92561 | 21.31318 | 21.39319 |
| 21.9529 | 29.47222 | 21.48915 | 21.93011 | 21.32535 | 21.4254 |
| 21.95689 | 30.67531 | 21.50078 | 21.93941 | 21.3426 | 21.44471 |
| 21.96146 | 30.87849 | 21.50826 | 21.94071 | 21.36772 | 21.46119 |
| 21.97654 |  | 21.51618 | 21.94427 | 21.37868 | 21.46564 |
| 21.97922 |  | 21.54317 | 21.97538 | 21.38649 | 21.48329 |
| 21.9911 |  | 21.55736 | 22.00391 | 21.4025 | 21.49322 |
| 21.99225 |  | 21.6062 | 22.02293 | 21.41016 | 21.5067 |
| 21.99768 |  | 21.61556 | 22.03833 | 21.41789 | 21.51188 |
| 22.00528 |  | 21.61563 | 22.04302 | 21.47781 | 21.52069 |
| 22.01447 |  | 21.6255 | 22.05776 | 21.48292 | 21.5238 |
| 22.0165 |  | 21.6308 | 22.07796 | 21.5067 | 21.52705 |
| 22.01939 |  | 21.63301 | 22.09058 | 21.50774 | 21.52824 |
| 22.0553 |  | 21.64251 | 22.09685 | 21.51085 | 21.53423 |
| 22.06245 |  | 21.64619 | 22.09786 | 21.52025 | 21.5394 |
| 22.07334 |  | 21.68088 | 22.14342 | 21.52409 | 21.57774 |
| 22.08185 |  | 21.69681 | 22.14407 | 21.55544 | 21.58298 |
| 22.08654 |  | 21.71757 | 22.15514 | 21.56954 | 21.63522 |
| 22.08676 |  | 21.74065 | 22.16693 | 21.58851 | 21.63655 |
| 22.09368 |  | 21.76225 | 22.16772 | 21.60119 | 21.64067 |
| 22.10643 |  | 21.77154 | 22.17834 | 21.61114 | 21.64802 |
| 22.12242 |  | 21.79274 | 22.20769 | 21.6308 | 21.64957 |
| 22.12544 |  | 21.79764 | 22.21393 | 21.64442 | 21.6542 |
| 22.14824 |  | 21.80786 | 22.21615 | 21.64869 | 21.6578 |
| 22.19851 |  | 21.82625 | 22.21701 | 21.64905 | 21.68095 |
| 22.21637 |  | 21.83231 | 22.22024 | 21.66141 | 21.68271 |
| 22.22475 |  | 21.8326 | 22.2216 | 21.68852 | 21.69454 |
| 22.2395 |  | 21.83916 | 22.22847 | 21.69006 | 21.7073 |
| 22.25554 |  | 21.85862 | 22.22948 | 21.6938 | 21.71068 |
| 22.25711 |  | 21.90876 | 22.23879 | 21.6952 | 21.71222 |
| 22.26362 |  | 21.92467 | 22.24058 | 21.70488 | 21.71471 |
| 22.2667 |  | 21.95798 | 22.28249 | 21.70488 | 21.72967 |
| 22.31627 |  | 21.97849 | 22.28664 | 21.70767 | 21.73172 |
| 22.31698 |  | 21.99182 | 22.28828 | 21.72116 | 21.74212 |
| 22.3182 |  | 21.99797 | 22.30792 | 21.72285 | 21.75303 |
| 22.32084 |  | 22.02192 | 22.30978 | 21.73091 | 21.75837 |
| 22.32248 |  | 22.03609 | 22.3172 | 21.73172 | 21.76049 |
| 22.3244 |  | 22.05754 | 22.31912 | 21.74146 | 21.77359 |
| 22.33467 |  | 22.10881 | 22.36752 | 21.74768 | 21.77366 |
| 22.35099 |  | 22.11673 | 22.38097 | 21.75522 | 21.78317 |
| 22.35213 |  | 22.13652 | 22.3821 | 21.75566 | 21.79815 |
| 22.35683 |  | 22.1698 | 22.38538 | 21.77059 | 21.81611 |
| 22.35954 |  | 22.18409 | 22.39725 | 21.78573 | 21.86525 |
| 22.36794 |  | 22.23342 | 22.40422 | 21.79252 | 21.86933 |
| 22.38595 |  | 22.26512 | 22.41907 | 21.79303 | 21.87144 |
| 22.39441 |  | 22.27149 | 22.42099 | 21.80932 | 21.88061 |
| 22.39832 |  | 22.27385 | 22.42624 | 21.81195 | 21.8988 |
| 22.40052 |  | 22.27992 | 22.49097 | 21.84587 | 21.90258 |
| 22.4028 |  | 22.28121 | 22.49699 | 21.85126 | 21.9153 |
| 22.4045 |  | 22.28671 | 22.51673 | 21.856 | 21.9418 |
| 22.40813 |  | 22.35384 | 22.52076 | 21.85738 | 21.94746 |
| 22.41886 |  | 22.37819 | 22.52119 | 21.8849 | 21.95341 |
| 22.41942 |  | 22.38573 | 22.52705 | 21.89894 | 21.95341 |
| 22.42546 |  | 22.43284 | 22.56555 | 21.89952 | 21.95711 |
| 22.43391 |  | 22.43291 | 22.56914 | 21.90694 | 21.96356 |
| 22.44299 |  | 22.43696 | 22.58381 | 21.90832 | 21.96393 |
| 22.4547 |  | 22.43881 | 22.59291 | 21.974 | 21.97393 |
| 22.49197 |  | 22.46193 | 22.59636 | 21.97936 | 21.98385 |
| 22.49282 |  | 22.49678 | 22.63938 | 22.0029 | 22.01447 |
| 22.50471 |  | 22.50372 | 22.67171 | 22.00615 | 22.03876 |
| 22.52034 |  | 22.51291 | 22.67269 | 22.04996 | 22.04642 |
| 22.52628 |  | 22.55383 | 22.6729 | 22.05292 | 22.05104 |
| 22.52868 |  | 22.56484 | 22.67473 | 22.0561 | 22.05602 |
| 22.5582 |  | 22.57119 | 22.68701 | 22.06115 | 22.07363 |
| 22.57528 |  | 22.57359 | 22.69003 | 22.06367 | 22.11867 |
| 22.58833 |  | 22.57528 | 22.69754 | 22.08005 | 22.17152 |
| 22.58995 |  | 22.57881 | 22.72194 | 22.11226 | 22.18028 |
| 22.62531 |  | 22.58544 | 22.7403 | 22.11788 | 22.18452 |
| 22.62988 |  | 22.59375 | 22.7501 | 22.12479 | 22.21192 |
| 22.64465 |  | 22.59939 | 22.75835 | 22.13753 | 22.22468 |
| 22.65056 |  | 22.61151 | 22.76129 | 22.13976 | 22.22582 |
| 22.67719 |  | 22.63523 | 22.78828 | 22.1422 | 22.22711 |
| 22.6887 |  | 22.64332 | 22.80518 | 22.14882 | 22.22754 |
| 22.69059 |  | 22.64535 | 22.81942 | 22.15802 | 22.23249 |
| 22.70624 |  | 22.66328 | 22.88082 | 22.16132 | 22.24294 |
| 22.7307 |  | 22.66433 | 22.88138 | 22.167 | 22.24509 |
| 22.7314 |  | 22.66588 | 22.88924 | 22.17418 | 22.26383 |
| 22.75961 |  | 22.68126 | 22.89529 | 22.18897 | 22.29007 |
| 22.76353 |  | 22.68596 | 22.90092 | 22.19177 | 22.30028 |
| 22.77752 |  | 22.7211 | 22.91309 | 22.20583 | 22.30299 |
| 22.78423 |  | 22.73483 | 22.96758 | 22.22439 | 22.30485 |
| 22.80344 |  | 22.73567 | 22.96979 | 22.22511 | 22.31527 |
| 22.81921 |  | 22.74149 | 22.99481 | 22.23098 | 22.3326 |
| 22.82263 |  | 22.75632 | 23.00021 | 22.2375 | 22.34729 |
| 22.83407 |  | 22.78395 | 23.00083 | 22.23958 | 22.35341 |
| 22.83665 |  | 22.80351 | 23.01799 | 22.24631 | 22.36965 |
| 22.85415 |  | 22.8685 | 23.0247 | 22.24652 | 22.43306 |
| 22.86125 |  | 22.88799 | 23.07934 | 22.2564 | 22.4349 |
| 22.88715 |  | 22.89021 | 23.09852 | 22.2762 | 22.44086 |
| 22.91121 |  | 22.91038 | 23.11078 | 22.27792 | 22.44363 |
| 22.91587 |  | 22.91475 | 23.14713 | 22.27928 | 22.45002 |
| 22.91746 |  | 22.91677 | 23.16976 | 22.28514 | 22.46179 |
| 22.92281 |  | 22.93594 | 23.21418 | 22.28728 | 22.46866 |
| 22.94552 |  | 22.9571 | 23.21617 | 22.29021 | 22.48134 |
| 22.95398 |  | 23.01917 | 23.22865 | 22.31349 | 22.48467 |
| 22.96168 |  | 23.01958 | 23.28192 | 22.31413 | 22.53179 |
| 22.9849 |  | 23.03549 | 23.29573 | 22.3244 | 22.55129 |
| 22.99405 |  | 23.04834 | 23.35219 | 22.34544 | 22.56315 |
| 23.00395 |  | 23.05684 | 23.35287 | 22.34686 | 22.56512 |
| 23.02021 |  | 23.07479 | 23.35758 | 22.36901 | 22.56604 |
| 23.02629 |  | 23.10182 | 23.38551 | 22.38025 | 22.5695 |
| 23.03224 |  | 23.1185 | 23.39804 | 22.38758 | 22.58036 |
| 23.03466 |  | 23.12828 | 23.46708 | 22.39661 | 22.60214 |
| 23.03666 |  | 23.1302 | 23.53707 | 22.40372 | 22.60756 |
| 23.03818 |  | 23.15511 | 23.55168 | 22.43916 | 22.62031 |
| 23.04841 |  | 23.15553 | 23.56114 | 22.45221 | 22.65119 |
| 23.04924 |  | 23.1668 | 23.59646 | 22.45228 | 22.6618 |
| 23.06457 |  | 23.18034 | 23.6142 | 22.45931 | 22.67726 |
| 23.07058 |  | 23.215 | 23.63799 | 22.47787 | 22.67922 |
| 23.07879 |  | 23.24475 | 23.66721 | 22.50074 | 22.68926 |
| 23.08755 |  | 23.249 | 23.689 | 22.5011 | 22.69656 |
| 23.11223 |  | 23.26844 | 23.70338 | 22.5011 | 22.7089 |
| 23.11912 |  | 23.27699 | 23.72796 | 22.51157 | 22.71458 |
| 23.13516 |  | 23.27864 | 23.74218 | 22.52691 | 22.72348 |
| 23.14541 |  | 23.3025 | 23.74225 | 22.54196 | 22.73098 |
| 23.16412 |  | 23.36201 | 23.75881 | 22.5635 | 22.74226 |
| 23.17738 |  | 23.39028 | 23.76859 | 22.56357 | 22.75087 |
| 23.19043 |  | 23.39436 | 23.77395 | 22.57098 | 22.75737 |
| 23.19606 |  | 23.45948 | 23.79986 | 22.60193 | 22.76269 |
| 23.22515 |  | 23.46382 | 23.80869 | 22.60869 | 22.76962 |
| 23.22899 |  | 23.52353 | 23.8383 | 22.62742 | 22.76969 |
| 23.2542 |  | 23.52462 | 23.85939 | 22.63649 | 22.83317 |
| 23.28342 |  | 23.5276 | 23.88628 | 22.64557 | 22.86926 |
| 23.30031 |  | 23.546 | 23.89314 | 22.64704 | 22.86996 |
| 23.30352 |  | 23.55087 | 23.90294 | 22.66553 | 22.87574 |
| 23.31459 |  | 23.56932 | 23.91672 | 22.692 | 22.87671 |
| 23.31862 |  | 23.58337 | 23.92325 | 22.69452 | 22.88068 |
| 23.3363 |  | 23.58377 | 23.95697 | 22.70322 | 22.88395 |
| 23.35512 |  | 23.59653 | 23.95936 | 22.71998 | 22.88513 |
| 23.36569 |  | 23.63018 | 23.97198 | 22.73357 | 22.88611 |
| 23.41226 |  | 23.63685 | 23.98792 | 22.75807 | 22.89863 |
| 23.41253 |  | 23.64527 | 23.99881 | 22.77004 | 22.92629 |
| 23.46355 |  | 23.65268 | 24.08914 | 22.77661 | 22.92941 |
| 23.474 |  | 23.66472 | 24.09139 | 22.77766 | 22.93413 |
| 23.47617 |  | 23.71467 | 24.09899 | 22.7829 | 22.93573 |
| 23.47807 |  | 23.77261 | 24.10843 | 22.82773 | 22.93754 |
| 23.48417 |  | 23.79644 | 24.14388 | 22.83651 | 22.94219 |
| 23.50234 |  | 23.80019 | 24.18638 | 22.84579 | 22.94961 |
| 23.53227 |  | 23.80929 | 24.20645 | 22.85819 | 22.94996 |
| 23.59781 |  | 23.83629 | 24.22499 | 22.85833 | 22.98331 |
| 23.61036 |  | 23.85399 | 24.23741 | 22.86808 | 22.98386 |
| 23.61454 |  | 23.87367 | 24.27464 | 22.87803 | 22.98663 |
| 23.63644 |  | 23.88341 | 24.30564 | 22.89717 | 22.99377 |
| 23.64991 |  | 23.92591 | 24.32706 | 22.90426 | 23.00595 |
| 23.66916 |  | 23.92737 | 24.32837 | 22.91559 | 23.02699 |
| 23.67441 |  | 23.93622 | 24.37447 | 22.9494 | 23.04081 |
| 23.67488 |  | 23.93842 | 24.43463 | 22.95572 | 23.04634 |
| 23.73145 |  | 23.99522 | 24.44863 | 22.95787 | 23.05345 |
| 23.74526 |  | 24.00093 | 24.47942 | 22.96744 | 23.05525 |
| 23.7578 |  | 24.02155 | 24.4975 | 22.97208 | 23.05753 |
| 23.76229 |  | 24.02374 | 24.5451 | 22.9777 | 23.0801 |
| 23.77703 |  | 24.02765 | 24.55256 | 22.97811 | 23.08686 |
| 23.78078 |  | 24.03693 | 24.55515 | 22.98262 | 23.10127 |
| 23.79798 |  | 24.03918 | 24.56066 | 22.98947 | 23.10885 |
| 23.80594 |  | 24.0411 | 24.56261 | 22.99633 | 23.11588 |
| 23.82239 |  | 24.06116 | 24.56488 | 22.99924 | 23.11609 |
| 23.82761 |  | 24.0781 | 24.57194 | 23.00145 | 23.11891 |
| 23.82774 |  | 24.08015 | 24.6071 | 23.00831 | 23.12525 |
| 23.86827 |  | 24.14084 | 24.62256 | 23.01433 | 23.13392 |
| 23.91732 |  | 24.2027 | 24.66192 | 23.02256 | 23.13427 |
| 23.92038 |  | 24.20408 | 24.69063 | 23.04067 | 23.16233 |
| 23.97119 |  | 24.20481 | 24.7208 | 23.05097 | 23.187 |
| 23.98746 |  | 24.24943 | 24.72479 | 23.05325 | 23.20444 |
| 24.01148 |  | 24.25633 | 24.73258 | 23.06623 | 23.20478 |
| 24.04256 |  | 24.26794 | 24.7607 | 23.07141 | 23.20601 |
| 24.05276 |  | 24.2846 | 24.76507 | 23.08245 | 23.20622 |
| 24.06626 |  | 24.36323 | 24.77285 | 23.08307 | 23.22392 |
| 24.08087 |  | 24.37571 | 24.78185 | 23.09479 | 23.22426 |
| 24.08683 |  | 24.38289 | 24.79823 | 23.1063 | 23.28342 |
| 24.11154 |  | 24.39491 | 24.8918 | 23.11195 | 23.30202 |
| 24.11451 |  | 24.43489 | 24.90127 | 23.1278 | 23.30571 |
| 24.12692 |  | 24.46914 | 24.90702 | 23.12965 | 23.32108 |
| 24.14671 |  | 24.47096 | 24.91278 | 23.13255 | 23.33145 |
| 24.15416 |  | 24.51978 | 24.91521 | 23.15147 | 23.33309 |
| 24.15957 |  | 24.53134 | 24.95952 | 23.15965 | 23.34196 |
| 24.16543 |  | 24.54218 | 24.96494 | 23.16384 | 23.34933 |
| 24.17169 |  | 24.58347 | 24.96992 | 23.16467 | 23.35744 |
| 24.19178 |  | 24.62871 | 24.99879 | 23.1894 | 23.36337 |
| 24.20941 |  | 24.65075 | 25.0906 | 23.2067 | 23.37332 |
| 24.25344 |  | 24.66799 | 25.10564 | 23.2161 | 23.38054 |
| 24.27037 |  | 24.68302 | 25.11864 | 23.2233 | 23.39198 |
| 24.27634 |  | 24.68309 | 25.16076 | 23.23016 | 23.393 |
| 24.28592 |  | 24.68522 | 25.16202 | 23.23242 | 23.40647 |
| 24.2911 |  | 24.6925 | 25.16367 | 23.23783 | 23.43455 |
| 24.29136 |  | 24.69386 | 25.17423 | 23.24098 | 23.43917 |
| 24.31174 |  | 24.69856 | 25.1915 | 23.24879 | 23.44393 |
| 24.32018 |  | 24.73323 | 25.2521 | 23.26399 | 23.44821 |
| 24.33151 |  | 24.74597 | 25.25803 | 23.27351 | 23.47 |
| 24.33707 |  | 24.76205 | 25.29621 | 23.28165 | 23.47074 |
| 24.38315 |  | 24.79964 | 25.37785 | 23.28719 | 23.47454 |
| 24.51082 |  | 24.9044 | 25.40406 | 23.29484 | 23.47509 |
| 24.54296 |  | 24.93546 | 25.41985 | 23.30202 | 23.48363 |
| 24.57239 |  | 24.96124 | 25.47654 | 23.31479 | 23.49224 |
| 24.57687 |  | 24.99044 | 25.51632 | 23.31923 | 23.51548 |
| 24.60697 |  | 25.02661 | 25.53074 | 23.32381 | 23.52719 |
| 24.6614 |  | 25.04563 | 25.63648 | 23.33166 | 23.53863 |
| 24.69005 |  | 25.08311 | 25.74056 | 23.34026 | 23.54661 |
| 24.70076 |  | 25.09371 | 25.76851 | 23.34435 | 23.56493 |
| 24.70379 |  | 25.10373 | 25.81321 | 23.36555 | 23.57411 |
| 24.70675 |  | 25.11895 | 25.88866 | 23.38994 | 23.58559 |
| 24.71771 |  | 25.12466 | 25.92192 | 23.39246 | 23.59437 |
| 24.73754 |  | 25.21456 | 25.92867 | 23.39708 | 23.59606 |
| 24.79521 |  | 25.26358 | 25.93156 | 23.3977 | 23.59727 |
| 24.8146 |  | 25.30842 | 26.00912 | 23.41158 | 23.60503 |
| 24.83872 |  | 25.42342 | 26.09213 | 23.42055 | 23.61245 |
| 24.84365 |  | 25.43757 | 26.14852 | 23.4281 | 23.62735 |
| 24.85314 |  | 25.46141 | 26.59031 | 23.42966 | 23.64763 |
| 24.85929 |  | 25.74155 | 26.83673 | 23.44277 | 23.67179 |
| 24.87939 |  | 25.79661 | 27.04149 | 23.45004 | 23.68154 |
| 24.89238 |  | 25.79772 | 27.15068 | 23.45989 | 23.70419 |
| 24.90159 |  | 25.86134 | 27.23026 | 23.47285 | 23.71876 |
| 24.94069 |  | 25.97218 | 27.28477 | 23.47902 | 23.74191 |
| 24.95984 |  | 26.10275 | 27.70494 | 23.50274 | 23.74788 |
| 24.97617 |  | 26.28294 | 27.7054 | 23.52177 | 23.76464 |
| 25.00178 |  | 26.29687 | 27.74928 | 23.52922 | 23.7943 |
| 25.04207 |  | 26.5109 | 27.88106 | 23.5487 | 23.79805 |
| 25.10291 |  | 26.51439 | 27.97667 | 23.56797 | 23.79932 |
| 25.10678 |  | 26.56922 | 28.03223 | 23.57114 | 23.81022 |
| 25.11198 |  | 26.62927 | 29.11612 | 23.58904 | 23.82319 |
| 25.13232 |  | 26.7169 | 30.83462 | 23.59342 | 23.84063 |
| 25.16677 |  | 26.79826 |  | 23.64897 | 23.86587 |
| 25.19434 |  | 26.82534 |  | 23.65012 | 23.8662 |
| 25.20028 |  | 26.83952 |  | 23.66122 | 23.88921 |
| 25.23261 |  | 26.86472 |  | 23.67697 | 23.89327 |
| 25.2504 |  | 27.03884 |  | 23.67945 | 23.89454 |
| 25.26786 |  | 27.09872 |  | 23.69707 | 23.90407 |
| 25.33828 |  | 27.12075 |  | 23.70164 | 23.9052 |
| 25.3727 |  | 27.1573 |  | 23.71198 | 23.92364 |
| 25.38481 |  | 27.81713 |  | 23.73614 | 23.92923 |
| 25.40293 |  | 28.22933 |  | 23.74057 | 23.94866 |
| 25.41246 |  | 28.50897 |  | 23.76745 | 23.94899 |
| 25.45678 |  | 28.87007 |  | 23.78185 | 23.9759 |
| 25.48367 |  | 29.67993 |  | 23.78312 | 23.97677 |
| 25.51289 |  | 31.49598 |  | 23.78908 | 23.99137 |
| 25.521 |  |  |  | 23.79116 | 23.99748 |
| 25.55872 |  |  |  | 23.80775 | 24.00922 |
| 25.55879 |  |  |  | 23.80935 | 24.01691 |
| 25.58538 |  |  |  | 23.81016 | 24.02825 |
| 25.6404 |  |  |  | 23.8242 | 24.03587 |
| 25.67118 |  |  |  | 23.83121 | 24.0415 |
| 25.68836 |  |  |  | 23.87727 | 24.05421 |
| 25.75961 |  |  |  | 23.9084 | 24.05487 |
| 25.89537 |  |  |  | 23.95172 | 24.05653 |
| 25.96709 |  |  |  | 23.96395 | 24.09833 |
| 25.96789 |  |  |  | 23.98102 | 24.12534 |
| 25.98285 |  |  |  | 23.98666 | 24.12857 |
| 26.00594 |  |  |  | 23.98971 | 24.1289 |
| 26.00655 |  |  |  | 24.00464 | 24.16543 |
| 26.04571 |  |  |  | 24.01313 | 24.20375 |
| 26.10623 |  |  |  | 24.01592 | 24.21566 |
| 26.17792 |  |  |  | 24.02102 | 24.229 |
| 26.32925 |  |  |  | 24.0368 | 24.23235 |
| 26.37258 |  |  |  | 24.04329 | 24.23505 |
| 26.48128 |  |  |  | 24.06804 | 24.24149 |
| 26.4835 |  |  |  | 24.07208 | 24.24471 |
| 26.50003 |  |  |  | 24.07393 | 24.25094 |
| 26.52441 |  |  |  | 24.10249 | 24.25114 |
| 26.5474 |  |  |  | 24.11517 | 24.25114 |
| 26.59342 |  |  |  | 24.11887 | 24.2558 |
| 26.68255 |  |  |  | 24.12606 | 24.25882 |
| 26.85963 |  |  |  | 24.13273 | 24.29201 |
| 26.90405 |  |  |  | 24.13834 | 24.29713 |
| 27.12087 |  |  |  | 24.14454 | 24.31161 |
| 27.1549 |  |  |  | 24.14684 | 24.32189 |
| 27.68033 |  |  |  | 24.16504 | 24.32418 |
| 27.78867 |  |  |  | 24.17545 | 24.33367 |
| 27.91605 |  |  |  | 24.20059 | 24.36101 |
| 28.52907 |  |  |  | 24.20231 | 24.40013 |
| 28.88479 |  |  |  | 24.20467 | 24.40026 |
|  |  |  |  | 24.20625 | 24.4556 |
|  |  |  |  | 24.22526 | 24.46947 |
|  |  |  |  | 24.22841 | 24.4722 |
|  |  |  |  | 24.22946 | 24.49633 |
|  |  |  |  | 24.2336 | 24.53958 |
|  |  |  |  | 24.26539 | 24.54652 |
|  |  |  |  | 24.27444 | 24.57375 |
|  |  |  |  | 24.28841 | 24.5814 |
|  |  |  |  | 24.33216 | 24.60225 |
|  |  |  |  | 24.33805 | 24.61066 |
|  |  |  |  | 24.34132 | 24.61758 |
|  |  |  |  | 24.34512 | 24.62948 |
|  |  |  |  | 24.34636 | 24.63181 |
|  |  |  |  | 24.38962 | 24.67805 |
|  |  |  |  | 24.39412 | 24.68876 |
|  |  |  |  | 24.39621 | 24.70533 |
|  |  |  |  | 24.4045 | 24.75369 |
|  |  |  |  | 24.41572 | 24.77163 |
|  |  |  |  | 24.44479 | 24.77607 |
|  |  |  |  | 24.44935 | 24.85006 |
|  |  |  |  | 24.48989 | 24.8516 |
|  |  |  |  | 24.55405 | 24.86121 |
|  |  |  |  | 24.60393 | 24.90133 |
|  |  |  |  | 24.60853 | 24.92211 |
|  |  |  |  | 24.62269 | 24.95231 |
|  |  |  |  | 24.63899 | 24.9798 |
|  |  |  |  | 24.65249 | 24.98688 |
|  |  |  |  | 24.66043 | 25.00389 |
|  |  |  |  | 24.66747 | 25.01051 |
|  |  |  |  | 24.67005 | 25.01382 |
|  |  |  |  | 24.6725 | 25.04099 |
|  |  |  |  | 24.68347 | 25.05993 |
|  |  |  |  | 24.68412 | 25.06781 |
|  |  |  |  | 24.69218 | 25.09333 |
|  |  |  |  | 24.69953 | 25.11661 |
|  |  |  |  | 24.70379 | 25.122 |
|  |  |  |  | 24.71403 | 25.14265 |
|  |  |  |  | 24.71809 | 25.18492 |
|  |  |  |  | 24.72737 | 25.19042 |
|  |  |  |  | 24.74539 | 25.25097 |
|  |  |  |  | 24.7508 | 25.26289 |
|  |  |  |  | 24.75594 | 25.33627 |
|  |  |  |  | 24.76565 | 25.36705 |
|  |  |  |  | 24.76829 | 25.37389 |
|  |  |  |  | 24.77755 | 25.37703 |
|  |  |  |  | 24.80523 | 25.38111 |
|  |  |  |  | 24.80934 | 25.44101 |
|  |  |  |  | 24.81178 | 25.4459 |
|  |  |  |  | 24.81858 | 25.46304 |
|  |  |  |  | 24.84833 | 25.4806 |
|  |  |  |  | 24.88368 | 25.49347 |
|  |  |  |  | 24.92102 | 25.49397 |
|  |  |  |  | 24.93757 | 25.5336 |
|  |  |  |  | 24.93974 | 25.53741 |
|  |  |  |  | 24.96092 | 25.55517 |
|  |  |  |  | 24.96749 | 25.56626 |
|  |  |  |  | 24.99395 | 25.57554 |
|  |  |  |  | 25.01013 | 25.57896 |
|  |  |  |  | 25.0191 | 25.61455 |
|  |  |  |  | 25.05586 | 25.63928 |
|  |  |  |  | 25.10088 | 25.64021 |
|  |  |  |  | 25.10189 | 25.64207 |
|  |  |  |  | 25.18062 | 25.72373 |
|  |  |  |  | 25.22687 | 25.74371 |
|  |  |  |  | 25.31446 | 25.74594 |
|  |  |  |  | 25.34394 | 25.78827 |
|  |  |  |  | 25.39591 | 25.81629 |
|  |  |  |  | 25.39754 | 25.81863 |
|  |  |  |  | 25.3998 | 25.84416 |
|  |  |  |  | 25.45584 | 25.84945 |
|  |  |  |  | 25.48348 | 25.86436 |
|  |  |  |  | 25.54152 | 25.99902 |
|  |  |  |  | 25.60311 | 26.01763 |
|  |  |  |  | 25.61275 | 26.09921 |
|  |  |  |  | 25.63586 | 26.19197 |
|  |  |  |  | 25.64176 | 26.2286 |
|  |  |  |  | 25.64723 | 26.3005 |
|  |  |  |  | 25.67639 | 26.33759 |
|  |  |  |  | 25.71661 | 26.36454 |
|  |  |  |  | 25.80198 | 26.40757 |
|  |  |  |  | 25.81 | 26.4672 |
|  |  |  |  | 25.85019 | 26.52417 |
|  |  |  |  | 25.9017 | 26.65533 |
|  |  |  |  | 25.92855 | 26.71822 |
|  |  |  |  | 25.9894 | 26.73978 |
|  |  |  |  | 26.06417 | 26.77567 |
|  |  |  |  | 26.15583 | 26.77989 |
|  |  |  |  | 26.17348 | 26.86704 |
|  |  |  |  | 26.17968 | 26.9712 |
|  |  |  |  | 26.27046 | 27.0775 |
|  |  |  |  | 26.29796 | 27.08961 |
|  |  |  |  | 26.32132 | 27.09396 |
|  |  |  |  | 26.3518 | 27.11317 |
|  |  |  |  | 26.35663 | 27.19293 |
|  |  |  |  | 26.40787 | 27.21938 |
|  |  |  |  | 26.4098 | 27.27835 |
|  |  |  |  | 26.4186 | 27.3056 |
|  |  |  |  | 26.44108 | 27.36426 |
|  |  |  |  | 26.61455 | 27.42512 |
|  |  |  |  | 26.61605 | 27.47744 |
|  |  |  |  | 26.70367 | 27.54007 |
|  |  |  |  | 26.71917 | 27.54793 |
|  |  |  |  | 26.72614 | 27.83327 |
|  |  |  |  | 26.73067 | 27.95014 |
|  |  |  |  | 26.83661 | 27.97018 |
|  |  |  |  | 26.92612 | 28.08484 |
|  |  |  |  | 27.00454 | 28.16551 |
|  |  |  |  | 27.00643 | 28.2899 |
|  |  |  |  | 27.18385 | 28.7876 |
|  |  |  |  | 27.21921 | 29.20273 |
|  |  |  |  | 27.22722 | 29.75869 |
|  |  |  |  | 27.23705 | 30.08946 |
|  |  |  |  | 27.34027 | 30.74137 |
|  |  |  |  | 27.3649 | 31.07106 |
|  |  |  |  | 27.3927 |  |
|  |  |  |  | 27.56492 |  |
|  |  |  |  | 27.63387 |  |
|  |  |  |  | 27.69011 |  |
|  |  |  |  | 27.69683 |  |
|  |  |  |  | 27.70057 |  |
|  |  |  |  | 27.73039 |  |
|  |  |  |  | 27.73343 |  |
|  |  |  |  | 27.85512 |  |
|  |  |  |  | 27.93144 |  |
|  |  |  |  | 28.0454 |  |
|  |  |  |  | 28.30841 |  |
|  |  |  |  | 28.42932 |  |
|  |  |  |  | 28.60577 |  |
|  |  |  |  | 28.84524 |  |
|  |  |  |  | 29.11803 |  |
|  |  |  |  | 29.37892 |  |
|  |  |  |  | 29.42079 |  |
|  |  |  |  | 29.63526 |  |
|  |  |  |  | 29.96734 |  |
|  |  |  |  | 30.2677 |  |
|  |  |  |  | 30.90704 |  |
|  |  |  |  | 31.95683 |  |
| Motoneurons mean diameters were measured in single optical sections through the nucleolus. | | | | | |
